# Inositol pyrophosphate profiling reveals regulatory roles of IP6K2-dependent enhanced IP_7_ metabolism in the enteric nervous system

**DOI:** 10.1016/j.jbc.2023.102928

**Published:** 2023-01-19

**Authors:** Masatoshi Ito, Natsuko Fujii, Saori Kohara, Shuho Hori, Masayuki Tanaka, Christopher Wittwer, Kenta Kikuchi, Takatoshi Iijima, Yu Kakimoto, Kenichi Hirabayashi, Daisuke Kurotaki, Henning J. Jessen, Adolfo Saiardi, Eiichiro Nagata

**Affiliations:** 1 Support Center for Medical Research and Education, Tokai University, Isehara, Japan; 2 Department of Neurology, Tokai University School of Medicine, Isehara, Japan; 3 Institute of Organic Chemistry, University of Freiburg, Freiburg, Germany; 4 Laboratory of Chromatin Organization in Immune Cell Development, International Research Center for Medical Sciences, Kumamoto University, Kumamoto, Japan; 5 Department of Molecular Life Science, Tokai University School of Medicine, Isehara, Japan; 6 Department of Forensic Medicine, Tokai University School of Medicine, Isehara, Japan; 7 Department of Pathology, Tokai University School of Medicine, Isehara, Japan; 8 Medical Research Council Laboratory for Molecular Cell Biology, University College London, London, United Kingdom

**Keywords:** inositol pyrophosphate, diphosphoinositol pentakisphosphate, inositol hexakisphosphate kinase 2, hydrophilic interaction liquid chromatography–tandem mass spectrometry, enteric nervous system, CNS, central nervous system, ENS, enteric nervous system, GIT, gastrointestinal tract, HILIC, hydrophilic interaction liquid chromatography, HRP, horseradish peroxidase, IP, inositol polyphosphate, IP_6_, inositol hexakisphosphate, IP_7_, diphosphoinositol pentakisphosphate, IP_8_, bisdiphosphoinositol tetrakisphosphate, IP6K, inositol hexakisphosphate kinase, MS/MS, tandem mass spectrometry, PP-IP, inositol pyrophosphate, qPCR, quantitative PCR, scRNA-seq, single-cell RNA sequencing, SRM, selected reaction monitoring, TBS, Tris-buffered saline

## Abstract

Inositol pyrophosphates regulate diverse physiological processes; to better understand their functional roles, assessing their tissue-specific distribution is important. Here, we profiled inositol pyrophosphate levels in mammalian organs using an originally designed liquid chromatography–mass spectrometry (LC-MS) protocol and discovered that the gastrointestinal tract (GIT) contained the highest levels of diphosphoinositol pentakisphosphate (IP_7_) and its precursor inositol hexakisphosphate (IP_6_). Although their absolute levels in the GIT are diet dependent, elevated IP_7_ metabolism still exists under dietary regimens devoid of exogenous IP_7_. Of the major GIT cells, enteric neurons selectively express the IP_7_-synthesizing enzyme IP6K2. We found that *IP6K2*-knockout mice exhibited significantly impaired IP_7_ metabolism in the various organs including the proximal GIT. In addition, our LC-MS analysis displayed that genetic ablation of *IP6K2* significantly impaired IP_7_ metabolism in the gut and duodenal muscularis externa containing myenteric plexus. Whole transcriptome analysis of duodenal muscularis externa further suggested that IP6K2 inhibition significantly altered expression levels of the gene sets associated with mature neurons, neural progenitor/stem cells, and glial cells, as well as of certain genes modulating neuronal differentiation and functioning, implying critical roles of the IP6K2-IP_7_ axis in developmental and functional regulation of the enteric nervous system. These results collectively reveal an unexpected role of mammalian IP_7_—a highly active IP6K2-IP_7_ pathway is conducive to the enteric nervous system.

Myo-inositol phosphates (IPs) are ubiquitously synthesized in all organisms and are involved in pleiotropic biological processes, most importantly in intracellular signaling (1). Among the IP family, inositol hexakisphosphate (IP_6_) is the most abundant and serves as a precursor of inositol pyrophosphates (PP-IPs) possessing diphosphate moieties at specific carbon positions (2, 3, 4, 5). Diphosphoinositol pentakisphosphate (IP_7_) and bisdiphosphoinositol tetrakisphosphate (IP_8_) are the most well-characterized PP-IPs in mammals and yeasts, and they carry diphosphate moieties at the 5-position (5-IP_7_) and 1,5-positions (1,5-IP_8_) of the inositol ring, respectively (4, 6). Recent studies using mammalian cells have demonstrated that PP-IPs regulate phosphate flux, energy homeostasis, and posttranscriptional processes at the molecular level (7, 8, 9, 10, 11). In mammals, 5-IP_7_ is synthesized by three inositol hexakisphosphate kinases (IP6Ks) IP6K1, IP6K2, and IP6K3. IP6K1 and IP6K2 are expressed in most mammalian tissues, with the highest expression in the brain and testis, whereas IP6K3 expression is mainly restricted to the muscles (12, 13, 14). *In vivo* studies using *IP6K1*- or *IP6K2*-knockout mice suggest that PP-IPs contribute to the development and maintenance of neuronal cells (15, 16, 17). In addition to these *in vivo* mice studies, our as well as other research groups have shown that PP-IPs are pathophysiologically involved in the progression of obesity (18, 19), in cancer (20) and in neurodegenerative disorders such as Huntington’s disease (21), amyotrophic lateral sclerosis (22), and Alzheimer’s disease (23). Therefore, PP-IPs are currently being considered as potential therapeutic targets for several diverse human disorders (24, 25). However, we are unaware of any systematic studies that have directly and comprehensively analyzed PP-IP distribution in mammalian tissues, which could provide valuable insights into the effects of pharmacological interventions on the PP-IP system.

Over the past decade, extensive efforts have been made to develop analytical methods for detecting PP-IPs. Traditionally, PP-IPs have been studied using radioisotopic ^3^H-inositol labeling coupled with anion exchange chromatography (26), which allows sensitive detection of metabolically labeled PP-IPs from cultured cells. Electrophoretic separation and colorimetric visualization of PP-IPs (27) have also become alternative standard methods for distinguishing PP-IPs. However, PP-IPs in mammalian tissues can neither be radioisotopically labeled nor explicitly detected using colorimetric visualization. A mass spectrometric method coupled with capillary electrophoretic separation (capillary electrophoresis–mass spectrometry) (28) was recently reported for sensitive analysis of PP-IPs in biological samples at the isomer level. However, the instrument setup involved is complex and requires skillful handling, and this method is therefore rarely available in research institutes.

We recently developed an analytical method that directly detects mammalian-derived IP_7_ and its precursor IP_6_ using conventional liquid chromatography–tandem mass spectrometry (MS/MS) coupled with hydrophilic interaction liquid chromatography (HILIC) (29), enabling the previously impossible quantitation of PP-IPs in mammalian tissues. In this study, we analyzed PP-IP and their precursor IP_6_ levels in mammalian organs using a refined HILIC-MS/MS protocol. We found that IP_7_ was present at explicit levels in the mammalian central nervous system (CNS), where IP6Ks are highly expressed. Surprisingly, we also discovered that the highest IP_7_ production was observed in the gastrointestinal tract (GIT), even after depletion of dietary derived IP_7_. Of the major GIT cells, enteric neurons selectively expressed IP_7_-synthesizing enzyme IP6K2, which was revealed by assessment of single-cell RNA sequencing (scRNA-seq) data sets and confirmed by immunohistochemical detection. Our HILIC-MS/MS survey using *IP6K2*-knockout (*IP6K2*^−/−^) mice exhibited that IP6K2-dependent enhanced IP_7_ metabolism exists in the gut and duodenal muscularis externa where the myenteric plexus is located. We further performed whole transcriptome analysis of *IP6K2*-deficient and wildtype (WT) duodenal muscularis externa to define a physiological role of IP6K2-IP_7_ pathway in the enteric nervous system (ENS).

## Results

### Refinement of HILIC-MS/MS protocol for PP-IP analysis

Before investigating PP-IP metabolism in mammalian tissues, we improved our HILIC-MS/MS analysis protocol for unequivocal detection and more precise quantitation of PP-IPs. Medronic acid was shown to improve the chromatographic detection of phosphorylated compounds (30). Consistently, a form of this solvent additive optimized for HILIC analysis (InfinityLab deactivator additive, Agilent Technologies) significantly improved the chromatographic peak shapes of IP_6_ and IP_7_ (Fig. S1*A*) and achieved clear detection of low abundance (10 pmol) of IP_7_ (Fig. S1*B*). We also determined the selected reaction monitoring (SRM) conditions for IP_8_ (Table S1) by learning its fragmentation pattern (Fig. S1*D*) to quantitate IP_8_ simultaneously with IP_6_ and IP_7_ (Fig. S1*E*). To benchmark this method for the detection of endogenous PP-IPs, we analyzed HCT116 cells treated with NaF, which is known to increase intracellular PP-IPs level (31). Our HILIC-MS/MS analysis detected explicit IP_7_ and IP_8_ SRM peaks in NaF-treated cells (Fig. S1*F*). We also observed a dose-dependent reduction in IP_7_ level and the IP_7_/IP_6_ ratio in HCT116 cells treated with the IP6K inhibitor TNP (Fig. S2). Thus, our refined HILIC-MS/MS protocol achieved robust, sensitive, and reliable detection of endogenous IP_6_, IP_7_, and IP_8_ in biological samples.

### The mammalian gastrointestinal tract contains high levels of PP-IPs

**Figure 1:**
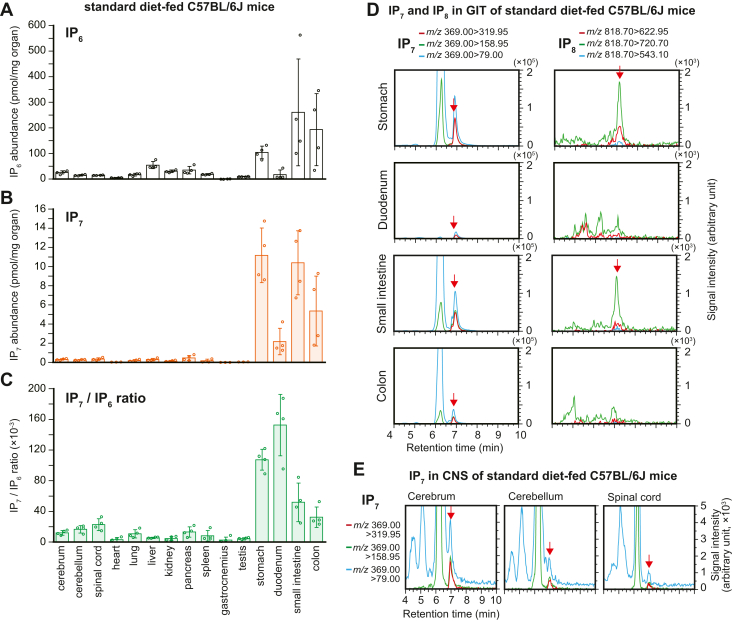
**The mammalian gastrointestinal tract (GIT) contains high levels of inositol polyphosphates.***A*–*C*, the concentrations of IP_6_ (*A*) and IP_7_ (*B*) and IP_7_/IP_6_ ratios (*C*) in the 15 organs of standard diet–fed male C57BL/6J mice. The values shown represent the mean ± standard deviation (SD) of four independent experiments and are expressed as pmol per mg of organ weight. *D*, representative SRM chromatograms of IP_7_ and IP_8_ in the GIT of standard diet–fed male C57BL/6J mice. The three best transitions per molecule are shown for the peak identification of each compound. The *arrows* indicate the SRM peaks of the corresponding analytes. *E*, representative SRM chromatograms of IP_7_ in the central nervous system (CNS) of standard diet–fed male C57BL/6J mice. The three best transitions per molecule are shown for the peak identification of each compound. *Arrows* indicate the SRM peak of corresponding analytes. SRM, selected reaction monitoring.

Using the newly developed HILIC-MS/MS protocol, we investigated the distribution of PP-IPs in experimental model rodents fed with a standard plant-based diet (CE-2; Clea, Japan). Fifteen organs, including the CNS and GIT, were harvested from standard diet–fed C57BL/6J male mice. Surprisingly, HILIC-MS/MS analysis showed that the GIT had the highest levels of IP_6_ and IP_7_, even after extensive rinsing of the organs with phosphate-buffered saline (PBS) to wash out the digested contents (Fig. 1, *A* and *B* andTable S2). Importantly, the IP_7_/IP_6_ ratio in the GIT was remarkably high, by far the highest in all organs examined (Fig. 1*C* and Table S2). A subtle IP_8_ SRM peak was detected in stomach and small intestine samples, wherein IP_7_ was abundant (Fig. 1*D*) but was not detected in other organs. While IP_7_ SRM peaks were clearly detected in CNS samples (Fig. 1*E*), IP_7_ levels in the CNS were modest compared with those in the GIT. Moreover, the IP_7_/IP_6_ ratio in the spinal cord appeared to be higher than that in the cerebrum (Fig. 1*C*).

**Figure 2:**
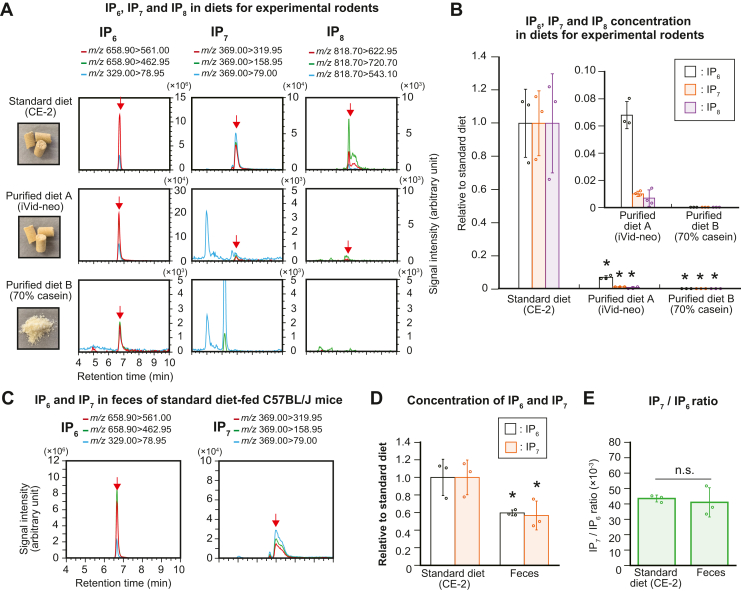
**Standard diet for experimental rodents contains high levels of inositol polyphosphates.***A*, photographs (leftmost) and SRM chromatograms of IP_6_, IP_7_, and IP_8_ (*right panels*) of standard diet (CE-2) and the two different purified diets (iVid-neo and 70% casein). *B*, relative concentrations of IP_6_, IP_7_, and IP_8_ in the standard and purified diets. The values shown represent the mean ± SD of three independent experiments and are expressed relative to those of the standard diet. *Asterisks* indicate statistical significance (*p*  <  0.05, Student’s *t* test) compared with the standard diet. *C*, representative SRM chromatograms of IP_6_ and IP_7_ in the feces of standard diet–fed C57BL/6J mice. *D*, relative concentrations of IP_6_ and IP_7_ in the feces of standard diet–fed male C57BL/6J mice. The values shown are expressed relative to those of the standard diet (n = 3). *Asterisks* indicate statistical significance (*p*  <  0.05, Student’s *t* test) compared with the standard diet. E. the IP_7_/IP_6_ ratio in standard diet and feces of standard diet–fed male C57BL/6J mice (n = 3). n.s., not significant (Student’s *t* test); SRM, selected reaction monitoring.

Several reports have shown that IPs (mainly IP_6_, known as phytic acid) are present in a variety of crop seeds (32, 33, 34, 35); moreover, plants also generate PP-IPs, which are crucial for phosphorus-starvation responses (36, 37, 38). Therefore, we assumed that the plant-based CE-2 diet contains IP_6_ and PP-IPs, and explicit chromatographic peaks of IP_6_, IP_7_, and IP_8_ were observed in CE-2 samples (Fig. 2*A*, upper panel). We next investigated their concentrations in purified diets with minimal levels of plant-derived components (Fig. 2*A*, middle and lower panels). The two purified diets examined (iVid-neo and 70% casein) contained low amounts of IP_6_ and negligible amounts of IP_7_ and IP_8_. Quantitative analysis revealed that the levels of all PP-IPs in both purified diets were less than 2% of those in CE-2 (Fig. 2*B*). The concentrations of IP_6_ and IP_7_ and the relative level of IP_8_ in CE-2 and two purified diets were summarized in Table S3. Thus, the abundances of IP_6_ and PP-IPs differ among different mouse diets, and standard mouse diet CE-2 substantially contains IP_6_ and PP-IPs, suggesting that dietary IP_6_ and IP_7_ affect the total levels of these molecules in the GIT of standard diet–fed mice.

Two possibilities were considered to explain the high IP_7_/IP_6_ ratios in the stomach and duodenum (Fig. 1*C*): increased absorption of dietary IP_7_ or the presence of endogenous IP_7_ in these two GIT organs. To verify the former possibility (selective intestinal absorption of IP_7_), we analyzed the feces of mice fed on CE-2 and estimated the loss of IP_6_ and IP_7_ in the digestive system. Similar to those for CE-2 samples, IP_6_ and IP_7_ SRM peaks were clearly observed in mouse feces samples (Fig. 2*C*), suggesting that considerable amounts of IP_6_ and IP_7_ still remain in the feces. Quantitative analysis showed that approximately 50% of IP_6_ and IP_7_ in ingested food remained in the feces (Fig. 2*D*). Since the IP_7_/IP_6_ ratio remained unchanged between undigested CE-2 and feces (Fig. 2*E*), dietary IP_6_ and IP_7_ might not be disproportionately degraded and absorbed throughout the digestive system. Therefore, we could exclude the possibility that dietary IP_7_ is preferentially absorbed in the GIT, further highlighting that the high IP_7_/IP_6_ ratios of the stomach and duodenum are probably attributable to cellular IP_7_ metabolism.

### Enhanced IP_7_ metabolism is retained in the proximal GIT of rodents under conditions of depleted dietary IP_6_ and PP-IP supply

**Figure 3:**
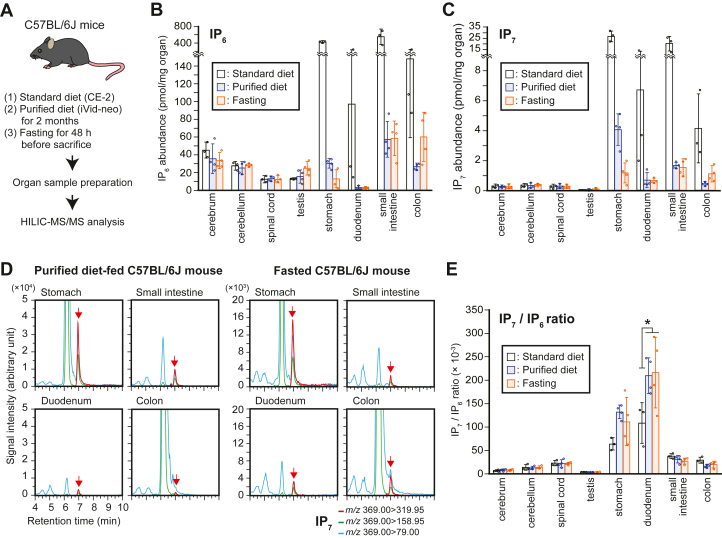
**Enhanced IP**_**7**_**metabolism is retained in the proximal GIT under depleted dietary PP-IP supply.***A*, schematic illustration of the experimental workflow. To precisely validate the presence of endogenously synthesized IP_6_ and PP-IPs in the GIT, C57BL/6J mice were maintained under two different conditions both of which remove dietary PP-IPs (*i.e*., 2 months feeding of purified diet iVid-neo and fasting for 48 h). CNS, testis, and GIT were harvested from these mice (each n = 4) and C57BL/6J mice fed a standard diet (n = 3) to compare tissue distribution of IP_6_ and PP-IPs. *B* and *C*. the concentrations of IP_6_ (*B*) and IP_7_ (*C*) in the CNS, testes, and GIT of male C57BL/6J mice under the three different conditions. The values shown are expressed as pmol per mg of organ weight. *D*, representative SRM chromatograms of IP_7_ in the GIT of male C57BL/6J mice fed with purified diet (*left panel*) or under fasting conditions (*right panel*). *Arrows* indicate the SRM peak of IP_7_. *E*, the IP_7_/IP_6_ ratios in the CNS, testes, and GIT of male C57BL/6J mice under the three different conditions. *Asterisk* indicates statistical significance (*p*  <  0.05, one-way ANOVA, Bonferroni-type post hoc test) compared with the standard diet–fed mice. CNS, central nervous system; GIT, gastrointestinal tract; HILIC, hydrophilic interaction liquid chromatography; MS/MS, tandem mass spectrometry; PP-IP, inositol pyrophosphates; SRM, selected reaction monitoring.

Dietary IP_6_ and PP-IPs present in the diet blur the direct detection of mammalian-derived IP_6_ and PP-IPs in the GIT of standard diet–fed mice. To more precisely validate the presence of endogenously synthesized IP_6_ and PP-IPs in the GIT, we prepared C57BL/6J mice maintained under two different conditions both of which remove dietary PP-IPs, namely, 2 months feeding of purified diet iVid-neo containing negligible amounts of PP-IPs (Fig. 2, *A* and *B* and Table S3) and fasting for 48 h, and compared their tissue concentrations of IP_6_ and PP-IPs with those from standard CE-2 diet–fed counterparts (Fig. 3*A*). The GIT of both purified diet–fed and fasted mice showed a reduction in IP_6_ and IP_7_ levels compared with those of standard diet–fed mice; however, the levels were still close to (in the case of IP_6_) or far greater than (in the case of IP_7_) the CNS levels (Fig. 3, *B* and *C*). IP_8_ was not detected in any of the tested organs of purified diet–fed and fasted mice. The SRM chromatograms of both purified diet–fed and fasted mice samples had explicit IP_7_ SRM peaks (Fig. 3*D*). Importantly, the stomach and duodenum of these mice showed prominently higher IP_7_/IP_6_ ratios than those of their standard diet–fed counterparts (Fig. 3*E*), implying further enhanced IP_7_ metabolism compensated for the overall reduced IP_7_ level. On the other hand, both purified diet–fed and fasted mice did not show any changes in the IP_6_ and IP_7_ levels as well as the IP_7_/IP_6_ ratio in the CNS and testis compared with those of mice fed a standard diet. However, as with standard diet–fed mice, both purified diet–fed and fasted mice showed higher IP_7_/IP_6_ ratios in the spinal cord than in the cerebrum, implying heterogeneous IP_7_ metabolic activity in the rostral and caudal CNS. We also investigated IP_7_ levels in the GIT of purified diet (70% casein)-fed Sprague–Dawley rats (Fig. S3*A*). Analogous to the results observed in the mouse model, both IP_6_ and IP_7_ levels in the GIT of these rats were drastically reduced compared with those in the standard diet–fed GIT and comparable with those in the CNS (Fig. S3, *B* and *C*). In addition, the IP_7_/IP_6_ ratio was higher in the stomach and duodenum of purified diet–fed rats compared with that of standard diet–fed rats (Fig. S3*D*), further demonstrating very active IP_7_ metabolism in the mammalian proximal GIT.

### Enteric neurons highly express IP6K2 in the mammalian GIT

**Figure 4:**
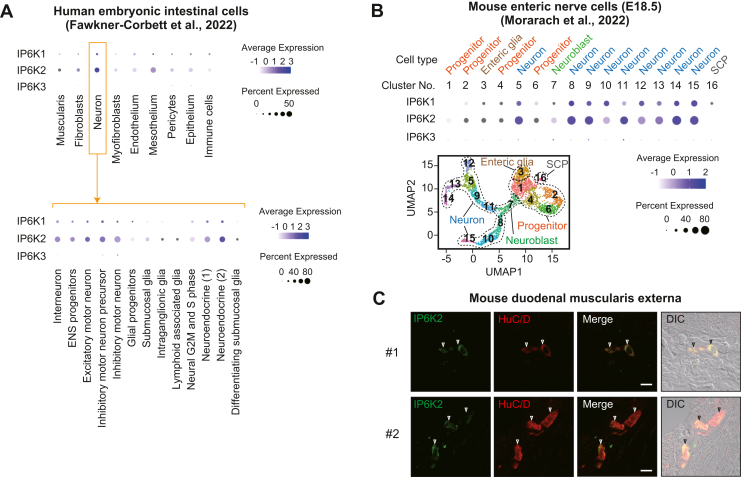
**Enteric neurons highly express IP6K2 in the mammalian GIT.***A*, expression analysis of IP6K1-3 in intestinal cell subsets using publicly available scRNA-seq datasets. Relative expression (log scale) of IP6K1-3 among human embryonic enteric cells (*upper panel*) and their neural cell subsets (*lower panel*), obtained by analysis of human embryonic intestinal cells scRNA-seq datasets, are shown (39). The size and color of the dots represent the percentage of cells that express IP6K1-3 mRNA and their average abundances within a cluster, respectively. *B*, UMAP-based unsupervised clustering of recently reported mouse embryonic (E18.5) ENS data (40) (*lower left panel*). Assignment of cell identities was based on the expression of signature genes as described in the literature: *Sox10* (Progenitor), *Ascl1* (Neuroblast), *Elavl4* (Neuron), *Plp1* (Enteric glia), and *Dhh* (SCP). Relative expression (log scale) of IP6K1-3 among the ENS clusters (*upper panel*) are shown. *C*, immunohistochemical analysis of IP6K2 expression in the duodenal muscularis externa of C57BL/6J mice. The neuronal marker HuC/D was also detected to identify enteric neurons in the myenteric plexuses. Open arrowheads indicate double-positive cells. Two different areas of confocal microscopy images are shown for clarity since enteric neurons exist sparsely on the GIT tissue sections. In addition, differential interference contrast images were overlaid onto the respective merged fluorescent images to identify cell contours and clarify the location where the double-positive cells exist in the GIT tissue. The scale bar represents 10 μm. E, embryonic day; ENS, enteric nervous system; GIT, gastrointestinal tract; SCP, Schwann cell precursor; UMAP, uniform manifold approximation and projection.

To investigate the expression levels of the three IP6Ks in each GIT cell type, we used scRNA-seq datasets and compared the expression levels of IP6Ks among GIT cell types. Quantitative analysis using a human embryonic intestinal cell scRNA-seq dataset (39) showed that enteric neural cells expressed the highest levels of IP6K2 among different intestinal cells (Fig. 4*A*, upper panel). In enteric neural cells, IP6K2 was selectively expressed across enteric neuron subsets, such as motor neurons, interneurons, and neuroendocrine cells but not in glial cells (Fig. 4*A*, lower panel). This analysis was further supported by the IP6K quantitation using both E15.5 (Fig. S4*A*) and E18.5 (Fig. 4*B*) mouse embryonic ENS scRNA-seq datasets (40). As in humans, IP6K2 isoform expression level in mouse enteric neurons was higher than in other neural cells such as neuroblasts, progenitors, glial cells, and Schwann cells. Moreover, the transcriptional analysis–based data were verified using immunohistochemical analyses. IP6K2 colocalized with the neuronal marker HuC/D in the mouse duodenal muscle layer, suggesting IP6K2 was expressed in the myenteric plexus (Fig. 4*C*). Other than enteric neurons, several cell types, including secretory progenitor cells, also expressed relatively high levels of IP6K2 (Fig. S4*B*). In addition, mouse enteric epithelial cell scRNA-seq data (41) showed that IP6K2 is expressed in mouse enteroendocrine cells (Fig. S4*C*). Expression levels of IP6K1 and IP6K3 in entire embryonic intestinal cells were low and negligible, respectively (Fig. 4, *A* and *B*). These results suggest that IP6K2 is highly expressed in mammalian enteric neurons.

### *IP6K2*^−/−^ mice show significant impairment of IP_7_ metabolism in the proximal GIT

**Figure 5:**
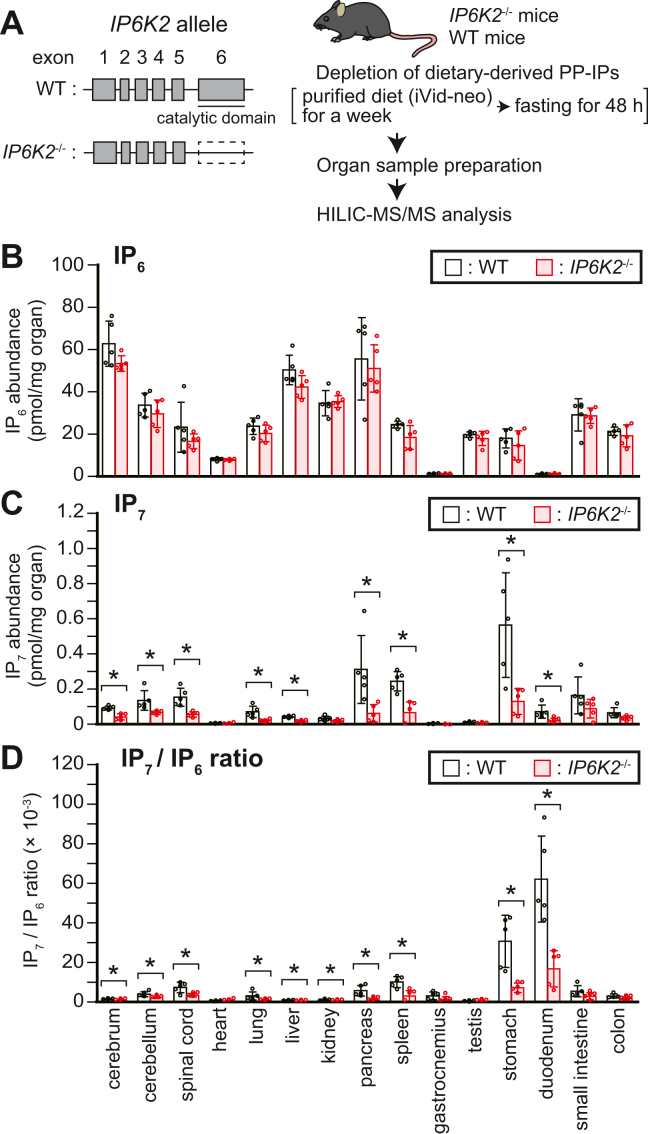
***IP6K2***^**−/−**^**mice show significant impairment of IP**_**7**_**metabolism in the proximal gastrointestinal tract.***A*, schematic depiction of the *IP6K2* genomic locus in *IP6K2*^−/−^ and WT mice (*left panel*) and the experimental workflow (*right panel*). IP6K2 exons and introns are represented as *boxes* and *lines*, respectively. *B*–*D*. the concentrations of IP_6_ (*B*) and IP_7_ (*C*), and IP_7_/IP_6_ ratios (*D*) in the central nervous system, gastrointestinal tract, and other organs of male *IP6K2*^−/−^ and WT mice. The values shown represent the mean ± SD of five independent experiments and are expressed as pmol per mg of organ weight. *Asterisks* indicate statistical significance (*p*  <  0.05, Student’s *t* test) compared with WT mice. HILIC, hydrophilic interaction liquid chromatography; MS/MS, tandem mass spectrometry.

To estimate the importance of IP6K2 in endogenous IP_7_ synthesis in the mammalian organs including GIT, we employed a genetically modified mouse in which *IP6K2* exon 6, encoding the kinase domain, was specifically deleted (Fig. 5*A*, left panel) (42). To avoid any contamination of dietary-derived IPs in our analysis, *IP6K2*-knockout (*IP6K2*^−/−^) or wildtype (WT) mice raised on the standard CE-2 diet were switched to a purified diet (iVid-neo) for 1 week and then fasted for 48 h before sacrifice (Fig. 5*A*, right panel). In WT mice, IP6K2 mRNA containing the exon 6 sequence was expressed in the proximal GIT but only marginally compared with the expression in the CNS (Fig. S5*A*). As expected, the IP6K2 transcript was absent in *IP6K2*^−/−^ mouse organs. We confirmed the loss of IP6K2 expression in *IP6K2*^−/−^ mice at the protein level using cerebrum lysate (Fig. S5*B*), because it has high IP6K2 protein expression and thus was useful for clearly validating the loss of IP6K2 in *IP6K2*^−/−^ mice. HILIC-MS/MS analysis showed that *IP6K2*^−/−^ mice had significantly lower levels of IP_7_ in various organs, including the stomach and duodenum, compared with those in their WT counterparts, while IP_6_ levels in each organ were almost the same between *IP6K2*^−/−^ and WT mice (Fig. 5, *B* and *C*). As previously observed (Figs. 3*E* and S3*D*), the IP_7_/IP_6_ ratios in the stomach and duodenum of WT mice were much higher than those in the other organs examined (Fig. 5*D*). These two organs of *IP6K2*^−/−^ mice exhibited significant reduction in the IP_7_/IP_6_ ratios compared with those of WT mice, suggesting active IP6K2 pathway works in stomach and duodenum. Consistently, the IP_7_ SRM peaks for the *IP6K2*^−/−^ mouse stomach and duodenum were smaller compared with those of WT mice, while IP_6_ levels were unchanged (Fig. S5, *C* and *D*). Collectively, these data demonstrate that IP6K2 is required for enhanced IP_7_ metabolism in the mammalian proximal GIT.

### IP6K2-dependent enhanced IP_7_ metabolism exists in the gut and duodenal muscularis externa where the myenteric plexus is located

**Figure 6:**
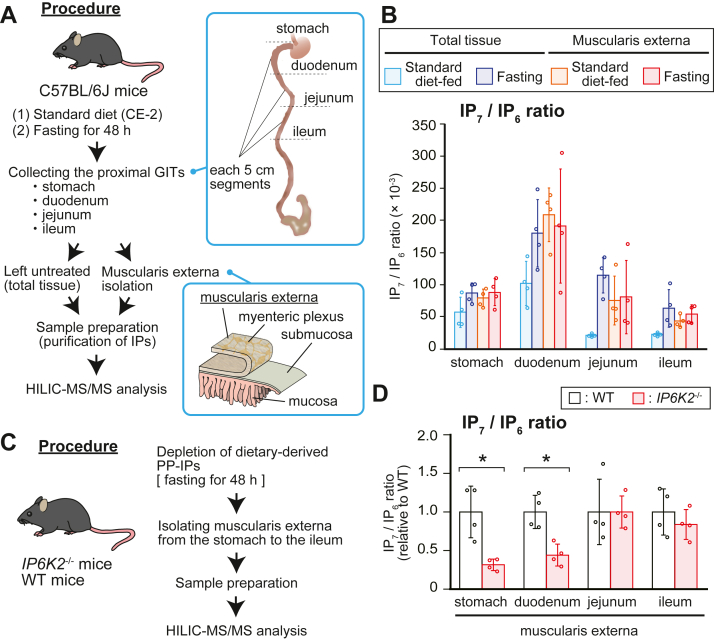
**IP6K2-dependent enhanced IP**_**7**_**metabolism exists in the gut and duodenal muscularis externa.***A*, schematic illustration of the experimental workflow. C57BL/6J mice were fed a standard diet or fasted for 48 h to deplete dietary IPs in the GIT. These mice were sacrificed to collect stomach and three consecutive 5-cm segments of the proximal GIT (duodenum, jejunum, ileum). The muscularis externa containing myenteric plexus as well as total tissues in the proximal GITs were subjected to HILIC-MS/MS analysis. *B*, the IP_7_/IP_6_ ratios in the muscularis externa and total tissue of four proximal GIT segments of C57BL/6J mice under the two different conditions. The values shown represent the mean ± SD of four independent experiments. *C*, schematic illustration of the experimental workflow. *IP6K2*^−/−^ and WT mice fasted for 48 h were sacrificed to collect four proximal GIT segments (stomach, duodenum, jejunum, ileum), which were then subjected to isolate muscularis externa. *D*, the IP_7_/IP_6_ ratios in the muscularis externa of the four GIT segments of *IP6K2*^−/−^ and WT mice. The values shown represent the mean ± SD of four independent experiments and are expressed relative to those for WT mice. *Asterisks* indicate statistical significance (*p*  <  0.05, Student’s *t* test) compared with WT mice. GIT, gastrointestinal tract; HILIC, hydrophilic interaction liquid chromatography; MS/MS, tandem mass spectrometry.

Since IP_7_-synthesizing kinase IP6K2 is selectively expressed in enteric neurons (Fig. 4), we next sought to investigate IP_7_ metabolism in the mammalian ENS. We collected the stomach and the consecutive 5-cm segments of duodenum, jejunum, and ileum from standard diet–fed (dietary PP-IPs provided) or fasted (dietary PP-IPs depleted) mice to more precisely survey the PP-IP profile in the proximal GIT. Some of these organs collected were subsequently used to isolate the muscularis externa where the myenteric plexus is located. These total GIT tissues and their muscularis externa were subjected to HILIC-MS/MS analysis to compare their IP_7_ metabolism (Fig. 6*A*). Similar to the results shown in Figure 3, 48 h fasting of mice rendered drastic reduction of IP_6_ and IP_7_ levels with concomitant increase of the IP_7_/IP_6_ ratio in total GIT tissues (Figs. 6*B* and S6). Although the muscularis externa contained less IP_6_ and IP_7_ than total GIT tissues, the muscle layer exhibited a higher IP_7_/IP_6_ ratio than total GIT tissues, which was less dependent on dietary conditions. The IP_7_/IP_6_ ratio of the duodenal muscularis externa was highest among the corresponding muscle layers of the neighboring GITs, implying highly active IP_7_ metabolism in the duodenal ENS. To verify the relationship between IP6K2-IP_7_ axis and the ENS, we first attempted to visualize the duodenal myenteric plexus of *IP6K2*^−/−^ mice by whole mount immunostaining (Fig. S7*A*). We found that *IP6K2* deletion largely affected neither the morphological features nor the neuronal cell density in the duodenal myenteric plexus (Fig. S7*B*). We next prepared the muscularis externa from the stomach to the ileum of WT and *IP6K2*^−/−^ mice, first depleting dietary IP_7_ in the GIT by 48 h fasting and performed HILIC-MS/MS analysis to evaluate IP_7_ metabolism in the ENS of *IP6K2*^−/−^ proximal GITs (Fig. 6*C*). While IP_6_ levels in the muscularis externa were almost equivalent between WT and *IP6K2*^−/−^ mice, IP_7_ levels and IP_7_/IP_6_ ratios were significantly reduced in the gut and duodenal muscularis externa of *IP6K2*^−/−^ mice (Figs. 6*D* and S8). These results suggest that IP6K2 actively produces IP_7_ in the gut and duodenal muscularis externa where enteric neurons are concentrated.

### The IP6K2-IP_7_ axis is crucial for certain neurotranscriptome profiles associated with ENS development and functioning

**Figure 7:**
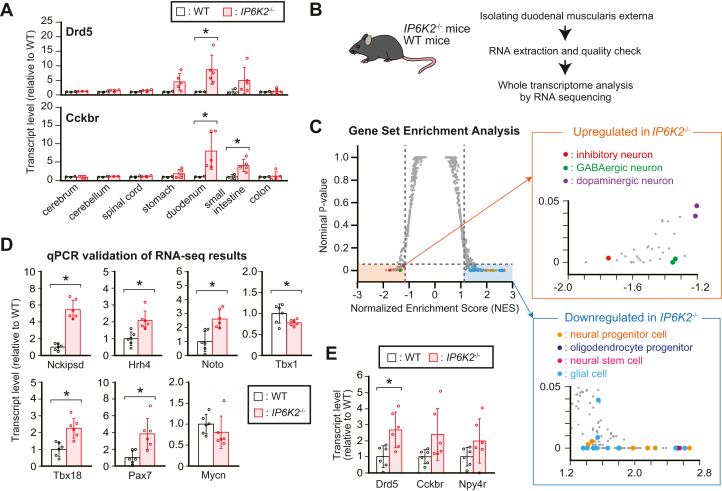
**IP6K2-IP**_**7**_**axis is crucial for certain neurotranscriptome profile associating with enteric nervous system development and functioning.***A*, transcript levels of two different neuronal genes (*Drd5* and *Cckbr*) in the CNS and GIT of *IP6K2*^−/−^ and WT mice. Data were normalized to 18S rRNA level. The values shown represent the mean ± SD of three (CNS of *IP6K2*^−/−^, and CNS and GIT of WT mice) and five (GIT of *IP6K2*^−/−^ mice) independent experiments and are expressed relative to those of WT mice. *Asterisks* indicate statistical significance (*p*  <  0.05, Student’s *t* test) compared with WT mice. *B*, schematic illustration of the experimental workflow. *IP6K2*^−/−^ and WT mice were sacrificed to collect the duodenal muscularis externa. High-quality total RNAs isolated from these tissues (each n =3) were subjected to whole transcriptome analysis by high-throughput RNA sequencing. *C*, Gene Set Enrichment Analysis of the enriched gene signature in *IP6K2*^−/−^ duodenal muscularis externa. Cell type signature gene sets (C8 in The Molecular Signatures Database ver7.5.1; http://www.gsea-msigdb.org/gsea/msigdb/index.jsp) were used for this analysis. Horizontal dashed line indicates nominal *p*-value 0.05, and vertical lines indicate normalized enriched score (NES) ± 1.2 cutoff. Gene sets assigned to neural progenitor cells and oligodendrocyte progenitor cells (source data are derived from Zhong *et al*. (79)), neural stem cells and glial cells (Fan *et al*. (80)), and mature neurons (inhibitory neurons, Cao *et al*. (81); GABAergic and dopaminergic neurons, La Manno *et al*. (82)) with nominal *p* < 0.05 and NES > 1.2 or < −1.2 are labeled in colored dots. *D*, qPCR validation of RNA-Seq results for seven neuronal genes significantly accumulated or depleted in *IP6K2*^−/−^ duodenal muscularis externa. qPCR data were normalized to β-actin level. The values shown represent the mean ± SD of six independent experiments and are expressed relative to those for WT mice. *Asterisks* indicate statistical significance (*p*  <  0.05, Student’s *t* test) compared with WT mice. *E*, transcript levels of three different neuronal genes (*Drd5*, *Cckbr* and *Npy4r*) in the duodenal muscularis externa of *IP6K2*^−/−^ and WT mice. Data were normalized to β-actin level. The values shown represent the mean ± SD of six independent experiments and are expressed relative to those for WT mice. *Asterisks* indicate statistical significance (*p*  <  0.05, Student’s *t* test) compared with WT mice. CNS, central nervous system; GIT, gastrointestinal tract.

Considering the active IP6K2-IP_7_ axis in the ENS, we assumed that alteration of IP_7_ metabolism by *IP6K2* deletion might affect the neuronal status in the proximal GIT. Thus, we randomly selected two neuronal genes expressed in the GIT as well as the CNS (43), namely, *dopamine receptor D5* (*Drd5*) and *cholecystokinin B receptor* (*Cckbr*), and investigated their mRNA levels in both the CNS and GIT by quantitative PCR (qPCR) (Fig. 7*A*). Compared with those in WT mice, these mRNA levels were explicitly increased from the stomach through the small intestine of *IP6K2*^−/−^ mice, especially in the duodenum, but not the colon and CNS. To comprehensively appreciate the role of IP6K2-dependent IP_7_ metabolism in neuronal gene expression in the mammalian ENS, we isolated the duodenal muscularis externa from WT and *IP6K2*^−/−^ mice and performed whole transcriptome analysis by RNA sequencing (RNA-Seq) (Fig. 7*B*). Gene set enrichment analysis showed that *IP6K2* deletion suppressed certain gene sets associated with neural stem/progenitor cells, oligodendrocyte progenitor cells, and glial cells, concomitantly with the induction of those of mature neurons such as inhibitory, dopaminergic, or GABAergic neurons (Figs. 7*C* and S9*A*), implying that inhibition of the IP6K2-IP_7_ pathway triggers neurodevelopmental imbalance in the mammalian ENS. The RNA-Seq analysis also exhibited that 107 and 134 of 23,405 genes were more than 1.5-fold increased or decreased in *IP6K2*^−/−^ with *p*-value less than 0.05, respectively (Fig. S9*B*). Pathway enrichment analysis of these genes showed that transcripts increased more than 1.5-fold in *IP6K2*^−/−^ were significantly enriched for proteins involved in neuronal signaling (neuroactive ligand-receptor interaction of Kyoto Encyclopedia of Genes and Genomes Annotation) (Fig. S9*C*). In these transcripts, we observed that seven genes associated with neuronal function (*Nckipsd*, and *Hrh4*) or development (*Noto*, *Tbx1*, *Tbx18*, *Pax7*, and *Mycn*) were prominently altered in their transcript levels between WT and *IP6K2*^−/−^ (Fig. S9*D*). To validate our RNA-Seq results, differential expression of these seven neuronal genes were further assessed by qPCR and all of these candidate genes exhibited similar significant or prominent changes in transcript levels as observed in RNA-Seq results (Fig. 7*D*). qPCR analysis also showed that expression of other neuronal genes, including *Drd5* and *Cckbr*, explicitly increased in *IP6K2*^−/−^ duodenal muscularis externa (Fig. 7*E*). These changes were not observed in the RNA-Seq analysis possibly because they were below the lower detection limit and/or because of quantitation error (44, 45). Collectively, the IP6K2-IP_7_ axis contributes to certain neurotranscriptome profiles involved in ENS development and functioning.

## Discussion

Mammalian PP-IPs have been implicated in obesity and diseases such as cancer and neurodegenerative disorders, and thus, their metabolism is a promising drug target (24, 5). For this reason, *in vivo* PP-IP profiling of mammalian tissues is an important subject of research. However, this objective has been thwarted by various technical difficulties. Recently, we developed an HILIC-MS/MS analysis protocol for the sensitive and specific detection of IP_7_ and its precursor IP_6_ (29). In this study, we quantified *in vivo* PP-IP levels in mammalian organs using a refined HILIC-MS/MS protocol and evaluated the contribution of IP6K2 to PP-IP metabolism by analyzing mice lacking this IP_7_-synthesizing kinase.

Surprisingly, we found that standard diet–fed rodents possess far more IP_7_ level in the GIT than in the CNS where IP_7_-synthesizing kinases IP6K1 and IP6K2 are most abundantly expressed among the mammalian organs (Fig. 1*B*). Since standard diet CE-2 contains abundant IP_6_ and PP-IPs (Fig. 2, *A* and *B* and Table S3), we deemed that dietary IPs should be taken away to precisely detect endogenous IP_7_ in the mammalian GIT. The rodent GIT in the two different conditions depleting dietary IP_7_ supply (*i.e*., feeding purified diets negligibly containing PP-IPs and fasting) still contained more abundant IP_7_ than that in the CNS (Figs. 3, *B* and *C* and S3, *B* and *C*). Furthermore, the IP_7_/IP_6_ ratio, an indicator of PP-IPs metabolism, was far higher in the proximal GIT than in the CNS irrespective of dietary condition (Figs. 3*E* and S3*D*), implying that endogenous IP_7_ synthesis is enhanced in the proximal GIT. In line with this result, IP_7_ levels in the stomach and duodenum were significantly diminished in *IP6K2*^−/−^ mice under dietary PP-IP-depleted conditions (Fig. 5*D*). Therefore, our HILIC-MS/MS analysis unexpectedly revealed enhanced IP_7_ metabolism in the mammalian GIT.

The GIT consists of several histological layers including the muscularis externa that contains the myenteric plexus, a collection of large neuronal assemblies in the GIT. Our HILIC-MS/MS survey of the proximal GIT clarified that the muscularis externa has a higher IP_7_/IP_6_ ratio than whole GIT tissues and the duodenal muscle layer has a much higher IP_7_/IP_6_ ratio than those of neighboring GIT segments (Fig. 6*B*). Considering the expression of the IP_7_-synthesizing enzyme IP6K2 in the myenteric plexus (Fig. 4) and the significant decrease of IP_7_/IP_6_ ratio in *IP6K2*-deficient gut and duodenal muscle layers (Fig. 6*D*), these observations lead to the idea that IP6K2 actively synthesizes endogenous IP_7_ in the ENS of the proximal GIT. Our results also implied the presence of endogenous IP_7_ in other GIT layers because total GIT tissues of dietary IP_7_-depleted (fasted) mice contained a greater amount of IP_7_ than their corresponding muscle layers (Fig. S6). Since another major nerve plexus exists in the submucosal layer (*i.e*., submucosal plexus), the submucosal layer may contain endogenous IP_7_ to some extent. This PP-IP might also exist in mucosal epithelium because certain enteroendocrine cells, including tuft cells, express IP6Ks at the relatively high level (Fig. S4, *B* and *C*). Tuft cell is one of rare cell types present in intestinal epithelium. Park *et al*. recently showed that tuft cell development is controlled by inositol polyphosphate multikinase, an enzyme responsible for driving IP metabolic pathway leading to IP_7_ synthesis (46). This fact and our data (Fig. S4, *B* and *C*) encourage to think that IP6K2 and IP_7_ might underlie tuft cell physiology as well. Future study is required for assessing cell type–specific IP_7_ metabolism to more precisely characterize IP_7_ metabolism in the GIT.

Although *IP6K2* was initially cloned as a Pi uptake stimulator from a rabbit duodenum complementary DNA library (47) and was annotated soon after as encoding an IP_7_-synthesizing enzyme (48, 49), the role of IP6K2 in the GIT has not been investigated until now. In this study, we demonstrated the presence of an active IP6K2-IP_7_ pathway in enteric neurons of the proximal GIT. Our RNA-Seq analysis of the duodenal muscularis externa indicated that genetic ablation of *IP6K2* causes certain gene products associated with mature neurons to accumulate concomitantly with the reduction of those of neural progenitor/stem cells and glial cells (Figs. 7*C* and S9*A*). Given that the developmental lineage of enteric neurons comprises several differentiation points such as neural crest cell migration, neuron–glia bifurcation, and neural stem/progenitor cell differentiation into mature enteric neurons (50), inhibition of the IP6K2-IP_7_ axis possibly causes developmental imbalances of the ENS at the several differentiation points including the maturation of both enteric neurons and glial cells. This idea is also supported by our findings that IP6K2 inhibition significantly altered the expression levels of several transcription factors regulating neural crest cell differentiation (Fig. 7*D*) (51, 52, 53, 54, 55, 56). In fact, IP6K2 activity was shown to be required for normal migration and development of neural crest cells in zebrafish (57). Besides, genetic inhibition of *IP6K2* in the duodenal muscularis externa significantly or prominently changed mRNA levels of several genes modulating neuronal functions (Fig. 7, *D* and *E*). Notably, *Nckipsd* transcript, one of the transcripts most significantly induced in *IP6K2*^−/−^ duodenal muscularis externa, contributes to the formation of neural dendrites (58, 59) and intracellular neuronal signaling (60). These pieces of knowledge lead to the hypothesis that the IP6K2-IP_7_ axis contributes to development and functions of enteric neurons, even though the axis does not largely affect the entire morphological output of the ENS (Fig. S7). The molecular mechanism whereby the IP6K2-IP_7_ axis regulates certain neurotranscriptome profile still remains elusive and will be clarified in future studies. Although developmental and functional ENS defects often result in fatal congenital disorders (61, 62), *IP6K2*^−/−^ mice do not show such severe phenotypic defects: *IP6K2*^−/−^ mice are born at Mendelian ratio and grow normally, similar to WT mice (42). Thus, the IP6K2-IP_7_ axis might serve as a fine-tuning factor for the developmental and functional regulation of the ENS, although we could not exclude the possibility that *IP6K1*, another major IP6K isoform, compensates for the loss of *IP6K2*. It will be meaningful to see whether ENS-specific inhibition of IP6K2 and/or IP6K1 influences gastrointestinal pathophysiologies and development of CNS diseases. Taken together, our observations provide valuable insights into the field of PP-IP biology and neurogastroenterology.

Since dysregulation of IP_7_ metabolism links to various human diseases including neurodegenerative diseases, studying IP_7_ metabolism in human organs provides essential knowledge from the clinical point of view. The refined HILIC-MS/MS protocol we described in this study is capable of detecting IP_6_ and IP_7_ not only in rodent organs but also in human postmortem organs dissected after forensic intervention (Fig. S10). Unlike in rodents, IP_7_ level and IP_7_/IP_6_ ratio in the human proximal GITs (esophagus, greater curvature and lesser curvature of the stomach) were less abundant compared with those in human CNS. This is probably due to the high turnover rate of PP-IPs and the delay in dissecting human postmortem organs. Forensic intervention and subsequent organ dissection take hours after death. The presence of the intestinal flora may also facilitate the decomposition of these molecules in the GITs (63). Thus, care should be taken to assess IP_7_ metabolism in human GITs. Although the refined HILIC-MS/MS protocol can detect both IP_7_ and IP_8_, this protocol failed to detect endogenous IP_8_ in all rodent and human organs examined in this study, even in mouse GIT where IP_7_ was explicitly abundant. This fact suggested that mammalian-derived IP_8_ is far less abundant than IP_7_ and its quantitative evaluation requires sample pooling or a more sensitive analytical protocol such as capillary electrophoresis–mass spectrometry (28). In any case, we demonstrated that our novel protocol was able to evaluate IP_7_ metabolism in human organs. Therefore, we foresee the diagnostic potential of our new analytical technique for analyzing IP_6_ and IP_7_ levels in clinical biopsy.

**Figure 8:**
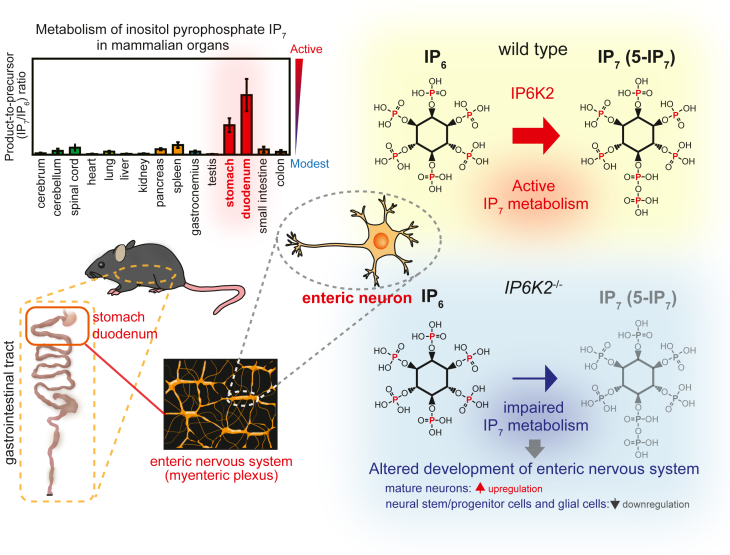
**Graphical summary of this study.** Among the major mammalian organs, stomach and duodenum possess the highest level of IP_7_/IP_6_ ratio, suggesting enhanced IP_7_ metabolism in the proximal gastrointestinal tract. This active metabolism retains in myenteric plexus wherein IP_7_-synthesizing enzyme IP6K2 is highly expressed. Ablation of *IP6K2* affects the configuration of neural cell types (*i.e*., inhibition of neural stem/progenitor cells and glial cells with concomitant induction of mature neurons) as well as expression levels of certain genes regulating enteric neuron development, demonstrating regulatory roles of active IP6K2-IP_7_ axis in the development of the enteric nervous system.

In conclusion, we investigated the distribution of PP-IPs in mammalian organs using a refined HILIC-MS/MS protocol and demonstrated that IP6K2-dependent IP_7_ metabolism was enhanced in the ENS of the proximal GIT. This finding was corroborated by the observation that impairment of IP6K2-dependent IP_7_ metabolism significantly altered certain neurotranscriptome profiles involved in ENS development (Fig. 8). Further studies are needed to dissect the role of IP_7_ in neurogastroenterology and processes involving the gut–brain axis. We believe that these findings shed new light on the physiological significance of the mammalian PP-IP pathway as well as the regulatory mechanisms of ENS development, which might contribute to a better understanding of human diseases associated with altered PP-IP metabolism.

## Experimental procedures

### Reagents and materials

LC-MS grade acetonitrile (Cat# 349672.5) and ammonium bicarbonate (Cat# 40867-50G) were purchased from Honeywell Burdick & Jackson. Ultrapure water (Cat# 210-01303) and formic acid (Cat# 067-04531) were obtained from Wako Pure Chemical Industries. Ultrapure grade ammonium hydroxide (28% w/v) was obtained from Kanto Chemical (Cat# 01266-3B). The InfinityLab deactivator additive was obtained from Agilent Technologies (Cat# 5191-3940). IP_6_ (Cat# 68388), sodium fluoride (Cat# S7920), and LC-MS grade medronic acid (Cat# 64255-1G-F) were purchased from Sigma-Aldrich. Hexadeutero-myo-inositol trispyrophosphate (ITPP-d_6_) was purchased from Toronto Research Chemicals (Cat# I666022). IP_7_ and IP_8_ were synthesized from myo-inositol using fluorenylmethyl phosphoramidite chemistry as described (64).

### Cell culture

HCT116 cells (RIKEN, RCB2979, RRID: CVCL_0291) were cultured in Dulbecco’s modified Eagle’s medium (Nacalai Tesque) supplemented with 10% fetal bovine serum in 5% CO_2_. Cells prepared at 60% confluence in 10-cm dishes were incubated for 1 h with or without 50 mM sodium fluoride (Sigma-Aldrich). After washing twice with PBS, the cells were lysed in cell lysis buffer (0.01% Triton X-100, 1 mM EDTA, 20 mM Tris-HCl). A small aliquot was set aside for protein quantitation, and the rest was used for purification of IPs.

### Mouse organs

All experiments involving animals were performed in accordance with protocols approved by institutional animal care guidelines (Tokai University School of Medicine). C57BL/6J mice and Sprague–Dawley rats obtained from Clea Japan (Tokyo, Japan) were maintained on a standard diet (CE-2; Clea Japan) or purified diet (iVid-neo; Oriental Kobo, 70% casein; Clea Japan). Some mice fed a standard diet or purified diet were fasted for 48 h before sacrifice. *IP6K2*^−/−^ (RRID: IMSR_JAX:036426) and WT mice maintained on a standard diet were switched to the purified diet for a week and subsequently fasted for 48 h before sacrifice. During fasting, mouse cages were changed to clean ones with new bedding every 24 h to reduce coprophagy. Mice and rats were anesthetized using isoflurane and then sacrificed by whole blood withdrawal from the left atrium. Before dissection of the organs, the animals were perfused transcardially with ice-cold PBS to wash out the residual blood and prevent the detection of IPs derived from blood cells. GIT organs (stomach, duodenum, small intestine, and colon) were cut open to remove feces and then extensively rinsed with PBS to wash out any dietary residuals. The duodenum, small intestine, and colon were harvested by cutting a 5-cm (mouse) or 10-cm (rat) segment from the distal end of stomach, between 10 and 15 cm (mouse) or 20 and 30 cm (rat) away from the duodenum and from the anus, respectively. The harvested organs were frozen until further use.

### Isolation of muscularis externa from mouse GITs

The muscularis externa containing myenteric plexuses was prepared from mouse GITs as previously described with some modification (65, 66). For HILIC-MS/MS analysis, mouse GIT segments were cut open along the attachment line of the mesentery and then placed onto a cold surface with the muscularis externa facing up. The muscularis externa of the GIT segments was isolated by gently scraping the outer layer with watchmaker tweezers under a binocular stereomicroscope. For whole-mount immunostaining, the mouse duodenum was cut open along the mesentery line, pinned onto a rubber plate, and then fixed with 4% paraformaldehyde overnight at 4 °C. The muscularis layer was then gently separated from the GIT segment using watchmaker tweezers and a cotton swab under a binocular stereomicroscope. For RNA extraction, mouse GITs were immersed in saturated ammonium sulfate solution containing 20 mM EDTA and 25 mM sodium citrate (pH5.2) to inhibit RNA degradation. The muscularis externa of the segments was placed over a glass rod and then peeled away using a cotton swab along the attachment line of the mesentery under a binocular stereomicroscope as described (67). Isolated muscularis externa was stored in saturated ammonium sulfate solution and then frozen until further use.

### Human postmortem organs

The human study was approved by the Ethics Committee of Tokai University (institutional review board number: 20I-02), and the study protocol conformed to the ethical guidelines of the 1975 Declaration of Helsinki (68). Written informed consent, allowing the experimental use of the organ samples, was obtained from the relatives of all subjects. Human postmortem organs were obtained at autopsies from three donated bodies (two men and one woman; mean age, 62.3 ± 22.8 years; average body mass index, 26.4 ± 6.6). Some anomalies, such as cardiac hypertrophy, were observed in their bodies by a forensic pathologist. To minimize organ decomposition, organ sampling was confined to cases where the death date and ambient temperature were explicit and the accumulated degree–days (environmental temperature [°C] × postmortem interval [day]) value—an index for evaluating the quality of forensic samples (69)—of all three bodies were very low (close to or less than 20). The harvested organ samples (approximately 400 mg) were frozen until further use.

### Gel electrophoresis of synthetic PP-IPs

The synthetic PP-IPs were validated using polyacrylamide gel electrophoresis as described (27). Briefly, synthetic PP-IPs samples mixed with orange G and bromophenol blue loading buffer were applied onto 35% polyacrylamide/Tris-borate-EDTA gel. The samples were electrophoresed overnight at 4 °C at 600 V and 6 mA until the orange G and bromophenol blue had run through two-thirds of the gel. Gels were stained with toluidine blue and scanned using a computer scanner.

### Purification of IPs

IPs in biological samples were purified as described (70), with some modification. Frozen organs, diets, and feces samples were homogenized using a Shake Master Neo (Bio Medical Science) in 500 μl of ultrapure water. Feces samples were air dried overnight before homogenization for accurate comparison of IP_6_ and IP_7_ concentrations with those in the diet. Crude lysate was mixed with an equal volume of 2 M perchloric acid, incubated on ice for 30 min, and centrifuged to remove tissue debris. After spiking with 3 nmol of ITPP-d_6_ as an internal control, 5 mg of titanium dioxide beads (GL Sciences, Cat# 5020-75000) were added to each sample. The beads were incubated at 4 °C for 30 min and washed twice with 1 M perchloric acid, and then 200 μl of 10% ammonium hydroxide was added for IP elution. The elution step was repeated to maximize recovery. The total eluate was dried using a SpeedVac concentrator (Thermo Fisher Scientific) and reconstituted in 125 μl of 100 mM ammonium carbonate/40% acetonitrile buffer, 50 μl of which was used for LC-MS.

### HILIC-MS/MS analysis for PP-IPs

Chromatographic experiments were performed using a Nexera UHPLC instrument (Shimadzu). HILIC-based chromatographic separation of IP_6_, IP_7_, IP_8_, and the internal control ITPP-d_6_ was achieved using a modified version of a previously described procedure (29). The mobile phase was composed of 300 mM ammonium bicarbonate buffer (pH 10.5) containing 0.1% InfinityLab deactivator additive (Agilent Technologies) as the aqueous mobile phase (eluent A), and 90% acetonitrile containing 10 mM ammonium bicarbonate buffer (pH 10.5) and 0.1% InfinityLab deactivator additive as the organic mobile phase (eluent B). Eluent B included more than 10% aqueous solvent to prevent polymeric aggregation of the major constituent (medronic acid) in the additive. In the entire LC system, chromatographic stainless steel tube was treated with 0.5% phosphoric acid in 90% acetonitrile overnight before analysis to block undesirable adsorption of analytes on the surface of the inner wall of the tube, while paying attention not to run the solvent into the mass spectrometer. The total flow rate of the mobile phase was 0.4 ml/min. Linear gradient separation was achieved as follows: 0 to 2 min, 75% B; 2 to 12 min, 75%–2% B; 12 to 15 min, 2% B.

### RNA extraction and quantitative PCR analysis

GIT segments and their muscularis externa were carefully collected and subjected to RNA extraction, as described (71). Total RNA was extracted using TRIzol reagent (Invitrogen, Cat# 15596026). RNA concentration and quality were determined using a NanoDrop 8000 spectrophotometer (Thermo Fisher Scientific) and the 4150 TapeStation system (Agilent Technologies), respectively. Complementary DNA was generated using the High-Capacity Reverse Transcription Kit (Applied Biosystems). qPCR was performed using the KAPA SYBR Fast qPCR kit (Kapa Biosystems, Cat# KK4602) and a StepOne Plus Real-Time PCR system (Applied Biosystems). The primer sequences used in this study are listed in Table S4.

### RNA sequencing

Total RNA samples of WT and *IP6K2*-deficient duodenal muscle externa with around 7.0 of RNA integrity number were subjected to RNA-Seq analysis. RNA sequencing libraries were prepared using TruSeq Stranded mRNA Kit (Illumina, Cat# 20020594) according to the manufacturer’s instructions. Each library was sequenced in 1 × 75 bp of single read mode using a NextSeq 500 platform (Illumina). Adapter sequences are removed from sequencing reads using Trim Galore (version 0.6.7; https://www.bioinformatics.babraham.ac.uk/projects/trim_galore/). Sequence reads were aligned to mouse genome (mm10) by HISAT2 (version 2.1.1) (72). Duplicate reads were removed using the MarkDuplicates module of the Picard package (version 2.27.3; http://broadinstitute.github.io/picard/). The following genes were excluded before processing for the expression data analysis: highly expressing mucosal digestive enzyme genes (*Amy2a1*, *Amy2a2*, *Amy2a3*, *Amy2a4*, *Amy2a5*, *Amy2b*, *Amy1*, *Try4*, *Try5*, *Try10*) contaminated during the muscularis isolation, mitochondrial genes, and long noncoding RNAs. Expression levels of genes annotated in GENCODE (version M25) were quantitated by TPMCalculator (version 0.0.3) (73). The software described above was run with the default parameters. Differentially expressed genes were identified by the EdgeR module of the TCC software (version 1.30.0) (74). Gene set enrichment analysis was performed as described (75). Gene sets used in this study were retrieved from The Molecular Signatures Database (version 7.5.1; http://www.gsea-msigdb.org/gsea/msigdb/index.jsp) (76). Pathway enrichment analysis was performed using the online database DAVID (http://david.abcc.ncifcrf.gov, RRID: SCR_001881) (77).

### Western blot analysis

Western blot analysis was performed as described (21). Membranes were incubated with anti-IP6K1 (Sigma-Aldrich, Cat# HPA040825, RRID: AB_10960426), anti-IP6K2 (Santa Cruz Biotechnology, Cat# sc-130012, RRID: AB_2127544), and anti-β-actin (Sigma-Aldrich, Cat# A5441, RRID: AB_476744) primary antibodies overnight at 4  °C. After rinsing 3 times in PBS containing 0.05% Tween-20, the membranes were incubated with the appropriate secondary antibodies conjugated with horseradish peroxidase (HRP) (donkey anti-rabbit IgG, HRP-linked F(ab’)_2_ fragment or sheep anti-mouse IgG, HRP-linked F(ab’)_2_ fragment; GE Healthcare). The immunoreactivities of the primary antibodies were visualized with Immobilon Western Chemiluminescent HRP Substrate (Millipore, Cat# WBKLS0500) and recorded using an Ez-Capture Analyzer (ATTO).

### Immunohistochemistry

Preparation of formalin-fixed, paraffin-embedded sections was performed as described (22). After deparaffinization and rehydration, the mouse tissue sections were incubated with Target Retrieval Solution (Dako, Cat# S1699) at 98 °C for 10 min. Thereafter, the sections were washed thrice with 0.05% Tween-20 in Tris-buffered saline (TBS), blocked using 5% normal goat serum for 15 min, and then incubated with primary antibodies against IP6K2 (Abcam, 1:100 dilution, Cat# ab179921) or HuC/D (Thermo Fisher Scientific, 1:100 dilution, Cat# A-21271, RRID: AB_221448) overnight at 4  °C. Rabbit immunoglobulin (Dako, Cat# X0936) and mouse IgG2b isotype control (Dako, Cat# X0944) were used to evaluate nonspecific binding. After rinsing thrice with 0.05% Tween-20 in TBS, the sections were incubated with secondary goat anti-rabbit IgG Alexa 488 (Thermo Fisher Scientific, 1:350 dilution, Cat# A-11070, RRID: AB_2534114) and goat anti-mouse IgG Alexa 594 (Thermo Fisher Scientific, 1:350 dilution, Cat# A-11020, RRID: AB_2534087) antibodies for 30 min at room temperature. The sections were then washed thrice with 0.05% Tween-20 in TBS and mounted using antifading medium (12.5 mg/ml DABCO, 90% glycerol, pH 8.8 in PBS). Confocal fluorescence images were obtained using an LSM 880 microscope (Carl Zeiss).

### Whole-mount immunostaining

Immunostaining of duodenal muscularis externa was performed as previously described with minor modifications (65, 66). Briefly, duodenal muscularis externa isolated from WT and *IP6K2*^−/−^ mice were blocked with 3% bovine serum albumin blocking solution containing the corresponding isotype control antibodies (IgG2a, R&D systems, Cat# MAB003; IgG2b, Dako) for 2 days after fixation with 4% paraformaldehyde overnight. The muscle layers were then washed with PBS containing 0.05% Triton X-100 and incubated with diluted primary antibodies against HuC/D (Thermo Fisher Scientific) or βIII-Tubulin (Biolegend, Cat# 801201, RRID: AB_2313773) for 3 days. After rinsing thrice with 0.05% Triton X-100 in PBS, the muscularis externa were then incubated with secondary goat anti-mouse IgG Alexa 594 (1:350, A-11020, Thermo Fisher Scientific) for 3 h at room temperature. The samples were then washed thrice with 0.05% Triton X-100 in PBS and mounted using antifading medium (12.5 mg/ml DABCO, 90% glycerol, pH 8.8 in PBS). Fluorescence images were obtained using an LSM 880 confocal microscope (Carl Zeiss).

### Computational analysis of scRNA-seq datasets

Publicly available human embryonic intestine scRNA-seq processed data (39) and mouse embryonic ENS matrix data (40) were downloaded from the Human Fetal Gut Atlas (https://simmonslab.shinyapps.io/FetalAtlasDataPortal/) and the GEO database (identifier: GSE149524), respectively. Mouse embryonic intestinal epithelial cell data (41) were obtained from the GEO database (identifier: GSE92332). The above datasets were analyzed using the R package Seurat version 4.0.0 (RRID: SCR_007322) (78) to perform dimensionality reduction by uniform manifold approximation and projection and/or generate dot plots showing the relative expression of IP6Ks across different clusters.

### Statistical analysis

Data are expressed as the mean ± SD. Differences between two or more groups were analyzed using two-tailed Student’s *t* test or one-way analysis of variance (ANOVA) followed by Bonferroni-type post hoc test, respectively. In RNA-Seq analyses, *p* values were determined using the corresponding analytical tools. Statistical significance was set at *p* < 0.05.

## Data availability

The raw RNA-Seq data have been deposited at the DNA Data Bank of Japan (DDBJ) and are publicly available with accession number DRA014733. Other individual datasets and corresponding files generated in this study are available upon reasonable request from the corresponding authors.

## Supporting information

This article contains supporting information.

## Conflict of interest

The authors declare that they have no conflicts of interest with the contents of this article.

## References

[1] Irvine R.F., Schell M.J. (2001). Back in the water: the return of the inositol phosphates. Nat. Rev. Mol. Cell Biol..

[2] Saiardi A. (2012). How inositol pyrophosphates control cellular phosphate homeostasis?. Adv. Biol. Regul..

[3] Wilson M.S., Livermore T.M., Saiardi A. (2013). Inositol pyrophosphates: between signalling and metabolism. Biochem. J..

[4] Shears S.B. (2015). Inositol pyrophosphates: why so many phosphates?. Adv. Biol. Regul..

[5] Shah A., Ganguli S., Sen J., Bhandari R. (2017). Inositol pyrophosphates: energetic, omnipresent and versatile signalling molecules. J. Indian Inst. Sci..

[6] Draskovic P., Saiardi A., Bhandari R., Burton A., Ilc G., Kovacevic M. (2008). Inositol hexakisphosphate kinase products contain diphosphate and triphosphate groups. Chem. Biol..

[7] Wilson M.S., Jessen H.J., Saiardi A. (2019). The inositol hexakisphosphate kinases IP6K1 and -2 regulate human cellular phosphate homeostasis, including XPR1-mediated phosphate export. J. Biol. Chem..

[8] Li X., Gu C., Hostachy S., Sahu S., Wittwer C., Jessen H.J. (2020). Control of XPR1-dependent cellular phosphate efflux by InsP_8_ is an exemplar for functionally-exclusive inositol pyrophosphate signaling. Proc. Natl. Acad. Sci. U. S. A..

[9] López-Sánchez U., Tury S., Nicolas G., Wilson M.S., Jurici S., Ayrignac X. (2020). Interplay between primary familial brain calcification-associated SLC20A2 and XPR1 phosphate transporters requires inositol polyphosphates for control of cellular phosphate homeostasis. J. Biol. Chem..

[10] Sahu S., Wang Z., Jiao X., Gu C., Jork N., Wittwer C. (2020). InsP_7_ is a small-molecule regulator of NUDT3-mediated mRNA decapping and processing-body dynamics. Proc. Natl. Acad. Sci. U. S. A..

[11] Gu C., Liu J., Liu X., Zhang H., Luo J., Wang H. (2021). Metabolic supervision by PPIP5K, an inositol pyrophosphate kinase/phosphatase, controls proliferation of the HCT116 tumor cell line. Proc. Natl. Acad. Sci. U. S. A..

[12] Saiardi A., Nagata E., Luo H.R., Snowman A.M., Snyder S.H. (2001). Identification and characterization of a novel inositol hexakisphosphate kinase. J. Biol. Chem..

[13] Moritoh Y., Oka M., Yasuhara Y., Hozumi H., Iwachidow K., Fuse H. (2016). Inositol hexakisphosphate kinase 3 regulates metabolism and lifespan in mice. Sci. Rep..

[14] Laha D., Portela-Torres P., Desfougères Y., Saiardi A. (2021). Inositol phosphate kinases in the eukaryote landscape. Adv. Biol. Regul..

[15] Fu C., Xu J., Cheng W., Rojas T., Chin A.C., Snowman A.M. (2017). Neuronal migration is mediated by inositol hexakisphosphate kinase 1 *via* alpha-actinin and focal adhesion kinase. Proc. Natl. Acad. Sci. U. S. A..

[16] Nagpal L., Fu C., Snyder S.H. (2018). Inositol hexakisphosphate kinase-2 in cerebellar granule cells regulates purkinje cells and motor coordination *via* protein 4.1N. J. Neurosci..

[17] Nagpal L., Kornberg M.D., Albacarys L.K., Snyder S.H. (2021). Inositol hexakisphosphate kinase-2 determines cellular energy dynamics by regulating creatine kinase-B. Proc. Natl. Acad. Sci. U. S. A..

[18] Chakraborty A., Koldobskiy M.A., Bello N.T., Maxwell M., Potter J.J., Juluri K.R. (2010). Inositol pyrophosphates inhibit Akt signaling, thereby regulating insulin sensitivity and weight gain. Cell.

[19] Ghoshal S., Zhu Q., Asteian A., Lin H., Xu H., Ernst G. (2016). TNP [N2-(m-Trifluorobenzyl), N6-(p-nitrobenzyl)purine] ameliorates diet induced obesity and insulin resistance *via* inhibition of the IP6K1 pathway. Mol. Metab..

[20] Rao F., Xu J., Fu C., Cha J.Y., Gadalla M.M., Xu R. (2015). Inositol pyrophosphates promote tumor growth and metastasis by antagonizing liver kinase B1. Proc. Natl. Acad. Sci. U. S. A..

[21] Nagata E., Saiardi A., Tsukamoto H., Okada Y., Itoh Y., Satoh T. (2011). Inositol hexakisphosphate kinases induce cell death in Huntington disease. J. Biol. Chem..

[22] Nagata E., Nonaka T., Moriya Y., Fujii N., Okada Y., Tsukamoto H. (2016). Inositol hexakisphosphate kinase 2 promotes cell death in cells with cytoplasmic TDP-43 aggregation. Mol. Neurobiol..

[23] Crocco P., Saiardi A., Wilson M.S., Maletta R., Bruni A.C., Passarino G. (2016). Contribution of polymorphic variation of inositol hexakisphosphate kinase 3 (IP6K3) gene promoter to the susceptibility to late onset Alzheimer's disease. Biochim. Biophys. Acta.

[24] Shears S.B. (2016). Towards pharmacological intervention in inositol pyrophosphate signalling. Biochem. Soc. Trans..

[25] Chakraborty A. (2018). The inositol pyrophosphate pathway in health and diseases. Biol. Rev. Camb. Philos. Soc..

[26] Azevedo C., Saiardi A. (2006). Extraction and analysis of soluble inositol polyphosphates from yeast. Nat. Protoc..

[27] Losito O., Szijgyarto Z., Resnick A.C., Saiardi A. (2009). Inositol pyrophosphates and their unique metabolic complexity: analysis by gel electrophoresis. PLoS One.

[28] Qiu D., Wilson M.S., Eisenbeis V.B., Harmel R.K., Riemer E., Haas T.M. (2020). Analysis of inositol phosphate metabolism by capillary electrophoresis electrospray ionization mass spectrometry. Nat. Commun..

[29] Ito M., Fujii N., Wittwer C., Sasaki A., Tanaka M., Bittner T. (2018). Hydrophilic interaction liquid chromatography-tandem mass spectrometry for the quantitative analysis of mammalian-derived inositol poly/pyrophosphates. J. Chromatogr. A..

[30] Hsiao J.J., Potter O.G., Chu T.W., Yin H. (2018). Improved LC/MS methods for the analysis of metal-sensitive analytes using medronic acid as a mobile phase additive. Anal. Chem..

[31] Menniti F.S., Miller R.N., Putney J.W., Shears S.B. (1993). Turnover of inositol polyphosphate pyrophosphates in pancreatoma cells. J. Biol. Chem..

[32] Dorsch J.A., Cook A., Young K., Anderson J.M., Bauman A.T., Volkmann C.J. (2003). Seed phosphorus and inositol phosphate phenotype of barley low phytic acid genotypes. Phytochemistry.

[33] Liu X., Villalta P.W., Sturla S.J. (2009). Simultaneous determination of inositol and inositol phosphates in complex biological matrices: quantitative ion-exchange chromatography/tandem mass spectrometry. Rapid Commun. Mass Spectrom..

[34] Kolozsvari B., Firth S., Saiardi A. (2015). Raman spectroscopy detection of phytic acid in plant seeds reveals the absence of inorganic polyphosphate. Mol. Plant.

[35] Duong Q.H., Clark K.D., Lapsley K.G., Pegg R.B. (2017). Quantification of inositol phosphates in almond meal and almond brown skins by HPLC/ESI/MS. Food Chem..

[36] Dong J., Ma G., Sui L., Wei M., Satheesh V., Zhang R. (2019). Inositol pyrophosphate InsP_8_ acts as an intracellular phosphate signal in arabidopsis. Mol. Plant.

[37] Ried M.K., Wild R., Zhu J., Pipercevic J., Sturm K., Broger L. (2021). Inositol pyrophosphates promote the interaction of SPX domains with the coiled-coil motif of PHR transcription factors to regulate plant phosphate homeostasis. Nat. Commun..

[38] Riemer E., Qiu D., Laha D., Harmel R.K., Gaugler P., Gaugler V. (2021). ITPK1 is an InsP_6_/ADP phosphotransferase that controls phosphate signaling in Arabidopsis. Mol. Plant.

[39] Fawkner-Corbett D., Antanaviciute A., Parikh K., Jagielowicz M., Gerós A.S., Gupta T. (2021). Spatiotemporal analysis of human intestinal development at single-cell resolution. Cell.

[40] Morarach K., Mikhailova A., Knoflach V., Memic F., Kumar R., Li W. (2021). Diversification of molecularly defined myenteric neuron classes revealed by single-cell RNA sequencing. Nat. Neurosci..

[41] Haber A.L., Biton M., Rogel N., Herbst R.H., Shekhar K., Smillie C. (2017). A single-cell survey of the small intestinal epithelium. Nature.

[42] Rao F., Cha J., Xu J., Hu R., Vandiver M.S., Tyagi R. (2014). Inositol pyrophosphates mediate the DNA-PK/ATM-p53 cell death pathway by regulating CK2 phosphorylation of Tti1/Tel2. Mol. Cell..

[43] Gremel G., Wanders A., Cedernaes J., Fagerberg L., Hallström B., Edlund K. (2015). The human gastrointestinal tract-specific transcriptome and proteome as defined by RNA sequencing and antibody-based profiling. J. Gastroenterol..

[44] Robert C., Watson M. (2015). Errors in RNA-Seq quantification affect genes of relevance to human disease. Genome Biol..

[45] Everaert C., Luypaert M., Maag J.L.V., Cheng Q.X., Dinger M.E., Hellemans J. (2017). Benchmarking of RNA-sequencing analysis workflows using whole-transcriptome RT-qPCR expression data. Sci. Rep..

[46] Park S.E., Lee D., Jeong J.W., Lee S.Y., Park S.J., Ryu J. (2022). Gut epithelial inositol polyphosphate multikinase alleviates experimental colitis *via* governing tuft cell homeostasis. Cell Mol. Gastroenterol. Hepatol..

[47] Norbis F., Boll M., Stange G., Markovich D., Verrey F., Biber J. (1997). Identification of a cDNA/protein leading to an increased Pi-uptake in Xenopus laevis oocytes. J. Membr. Biol..

[48] Saiardi A., Erdjument-Bromage H., Snowman A.M., Tempst P., Snyder S.H. (1999). Synthesis of diphosphoinositol pentakisphosphate by a newly identified family of higher inositol polyphosphate kinases. Curr. Biol..

[49] Schell M.J., Letcher A.J., Brearley C.A., Biber J., Murer H., Irvine R.F. (1999). PiUS (Pi uptake stimulator) is an inositol hexakisphosphate kinase. FEBS Lett..

[50] Rao M., Gershon M.D. (2018). Enteric nervous system development: what could possibly go wrong?. Nat. Rev. Neurosci..

[51] Knoepfler P.S., Cheng P.F., Eisenman R.N. (2002). N-myc is essential during neurogenesis for the rapid expansion of progenitor cell populations and the inhibition of neuronal differentiation. Genes Dev..

[52] Vitelli F., Morishima M., Taddei I., Lindsay E.A., Baldini A. (2002). Tbx1 mutation causes multiple cardiovascular defects and disrupts neural crest and cranial nerve migratory pathways. Hum. Mol. Genet..

[53] Abdelkhalek H.B., Beckers A., Schuster-Gossler K., Pavlova M.N., Burkhardt H., Lickert H. (2004). The mouse homeobox gene Not is required for caudal notochord development and affected by the truncate mutation. Genes Dev..

[54] Bussen M., Petry M., Schuster-Gossler K., Leitges M., Gossler A., Kispert A. (2004). The T-box transcription factor Tbx18 maintains the separation of anterior and posterior somite compartments. Genes Dev..

[55] Basch M., Bronner-Fraser M., García-Castro M.I. (2006). Specification of the neural crest occurs during gastrulation and requires Pax7. Nature.

[56] Simões-Costa M.S., McKeown S.J., Tan-Cabugao J., Sauka-Spengler T., Bronner M.E. (2012). Dynamic and differential regulation of stem cell factor FoxD3 in the neural crest is encrypted in the genome. PLoS Genet..

[57] Sarmah B., Wente S.R. (2010). Inositol hexakisphosphate kinase-2 acts as an effector of the vertebrate Hedgehog pathway. Proc. Natl. Acad. Sci. U. S. A..

[58] Fukuoka M., Suetsugu S., Miki H., Fukami K., Endo T., Takenawa T. (2001). A novel neural Wiskott-Aldrich syndrome protein (N-WASP) binding protein, WISH, induces Arp2/3 complex activation independent of Cdc42. J. Cell Biol..

[59] Lee S., Lee K., Hwang S., Kim S.H., Song W.K., Park Z.Y. (2006). SPIN90/WISH interacts with PSD-95 and regulates dendritic spinogenesis *via* an N-WASP-independent mechanism. EMBO J..

[60] Kim S.M., Choi K.Y., Cho I.H., Rhy J.H., Kim S.H., Park C.S. (2009). Regulation of dendritic spine morphology by SPIN90, a novel Shank binding partner. J. Neurochem..

[61] Furness J.B. (2012). The enteric nervous system and neurogastroenterology. Nat. Rev. Gastroenterol. Hepatol..

[62] Wright C.M., Garifallou J.P., Schneider S., Mentch H., Kothakapa D.R., Maguire B.A. (2021). Dlx1/2 mice have abnormal enteric nervous system function. JCI insight.

[63] Musshoff F., Klotzbach H., Block W., Traeber F., Schild H., Madea B. (2011). Comparison of post-mortem metabolic changes in sheep brain tissue in isolated heads and whole animals using 1H-MR spectroscopy--preliminary results. Int. J. Leg. Med..

[64] Pavlovic I., Thakor D.T., Vargas J.R., McKinlay C.J., Hauke S., Anstaett P. (2016). Cellular delivery and photochemical release of a caged inositol-pyrophosphate induces PH-domain translocation in cellulo. Nat. Commun..

[65] Fujita M., Yagi T., Okura U., Tanaka J., Hirashima N., Tanaka M. (2018). Calcineurin B1 deficiency in glial cells induces mucosal degeneration and inflammation in mouse small intestine. Biol. Pharm. Bull..

[66] Ahrends T., Weiner M., Mucida D. (2022). Isolation of myenteric and submucosal plexus from mouse gastrointestinal tract and subsequent flow cytometry and immunofluorescence. STAR Protoc..

[67] Smith T.H., Ngwainmbi J., Grider J.R., Dewey W.L., Akbarali H.I. (2013). An in-vitro preparation of isolated enteric neurons and glia from the myenteric plexus of the adult mouse. J. Vis. Exp..

[68] World Medical Association (2013). World medical association declaration of Helsinki: ethical principles for medical research involving human subjects. JAMA.

[69] Pittner S., Ehrenfellner B., Monticelli F.C., Zissler A., Sänger A.M., Stoiber W. (2016). Postmortem muscle protein degradation in humans as a tool for PMI delimitation. Int. J. Leg. Med..

[70] Wilson M.S., Bulley S.J., Pisani F., Irvine R.F., Saiardi A. (2015). A novel method for the purification of inositol phosphates from biological samples reveals that no phytate is present in human plasma or urine. Open Biol..

[71] Augereau C., Lemaigre F.P., Jacquemin P. (2016). Extraction of high-quality RNA from pancreatic tissues for gene expression studies. Anal. Biochem..

[72] Kim D., Paggi J.M., Park C., Bennett C., Salzberg S.L. (2018). Graph-based genome alignment and genotyping with HISAT2 and HISAT-genotype. Nat. Biotech..

[73] Vera Alvarez R., Pongor L.S., Mariño-Ramírez L., Landsman D. (2019). TPMCalculator: one-step software to quantify mRNA abundance of genomic features. Bioinformatics.

[74] Sun J., Nishiyama T., Shimizu K., Kadota K. (2013). Tcc: an R package for comparing tag count data with robust normalization strategies. BMC Bioinform..

[75] Subramanian A., Tamayo P., Mootha V.K., Mukherjee S., Ebert B.L., Gillette M.A. (2005). Gene set enrichment analysis: a knowledge-based approach for interpreting genome-wide expression profiles. Proc. Natl. Acad. Sci. U. S. A..

[76] Liberzon A., Birger C., Thorvaldsdóttir H., Ghandi M., Mesirov J.P., Tamayo P. (2015). The Molecular Signatures Database (MSigDB) hallmark gene set collection. Cell Syst..

[77] Huang da W., Sherman B.T., Lempicki R.A. (2009). Systematic and integrative analysis of large gene lists using DAVID bioinformatics resources. Nat. Protoc..

[78] Butler A., Hoffman P., Smibert P., Papalexi E., Satija R. (2018). Integrating single-cell transcriptomic data across different conditions, technologies, and species. Nat. Biotechnol..

[79] Zhong S., Zhang S., Fan X., Wu Q., Yan L., Dong J. (2018). A single-cell RNA-seq survey of the developmental landscape of the human prefrontal cortex. Nature.

[80] Fan X., Dong J., Zhong S., Wei Y., Wu Q., Yan L. (2018). Spatial transcriptomic survey of human embryonic cerebral cortex by single-cell RNA-seq analysis. Cell Res..

[81] Cao J., O’Day D.R., Pliner H.A., Kingsley P.D., Deng M., Daza R.M. (2020). A human cell atlas of fetal gene expression. Science.

[82] La Manno G., Gyllborg D., Codeluppi S., Nishimura K., Salto C., Zeisel A. (2016). Molecular diversity of midbrain development in mouse, human, and stem cells. Cell.

