# Supplementary material for: Inositol pyrophosphate profiling reveals regulatory roles of IP6K2-dependent enhanced IP7 metabolism in the enteric nervous system
Source: J Biol Chem. 2023 Jan 19;299(3):102928. doi: 10.1016/j.jbc.2023.102928 (PMC9957762; doi:10.1016/j.jbc.2023.102928)
Supplement: Supporting information figures [file mmc1.docx]

Supplementary Figures for:

**Inositol pyrophosphate profiling reveals regulatory roles of IP6K2-dependent enhanced IP_7_ metabolism**

**in the enteric nervous system**

Running title: IP6K2-IP_7_ axis regulates the enteric nervous system

Masatoshi Ito^1,*^, Natsuko Fujii^2^, Saori Kohara^2^, Shuho Hori^1^, Masayuki Tanaka^1^, Christopher Wittwer^3^, Kenta Kikuchi^4^, Takatoshi Iijima^5^, Yu Kakimoto^6^, Kenichi Hirabayashi^7^, Daisuke Kurotaki^4^, Henning J. Jessen^3^, Adolfo Saiardi^8^, Eiichiro Nagata^2,*^

^1^Support Center for Medical Research and Education, Tokai University, Isehara, Japan; ^2^Department of Neurology, Tokai University School of Medicine, Isehara, Japan; ^3^Institute of Organic Chemistry, University of Freiburg, Freiburg, Germany; ^4^Laboratory of Chromatin Organization in Immune Cell Development, International Research Center for Medical Sciences, Kumamoto University, Kumamoto, Japan; ^5^Department of Molecular Life Science, ^6^Department of Forensic Medicine, and ^7^Department of Pathology, Tokai University School of Medicine, Isehara, Japan; ^8^Medical Research Council Laboratory for Molecular Cell Biology, University College London, London, United Kingdom

* For correspondence:

Masatoshi Ito, [masa104-ito@tokai-u.jp](mailto:masa104-ito@tokai-u.jp); Eiichiro Nagata, [enagata@is.icc.u-tokai.ac.jp](mailto:enagata@is.icc.u-tokai.ac.jp)

Present address for Masatoshi Ito: Department of Legal Medicine at St. Marianna University School of Medicine, Kawasaki, Kanagawa 216-8511, Japan; e-mail: [masatoshi.ito@marianna-u.ac.jp](mailto:masatoshi.ito@marianna-u.ac.jp)

**
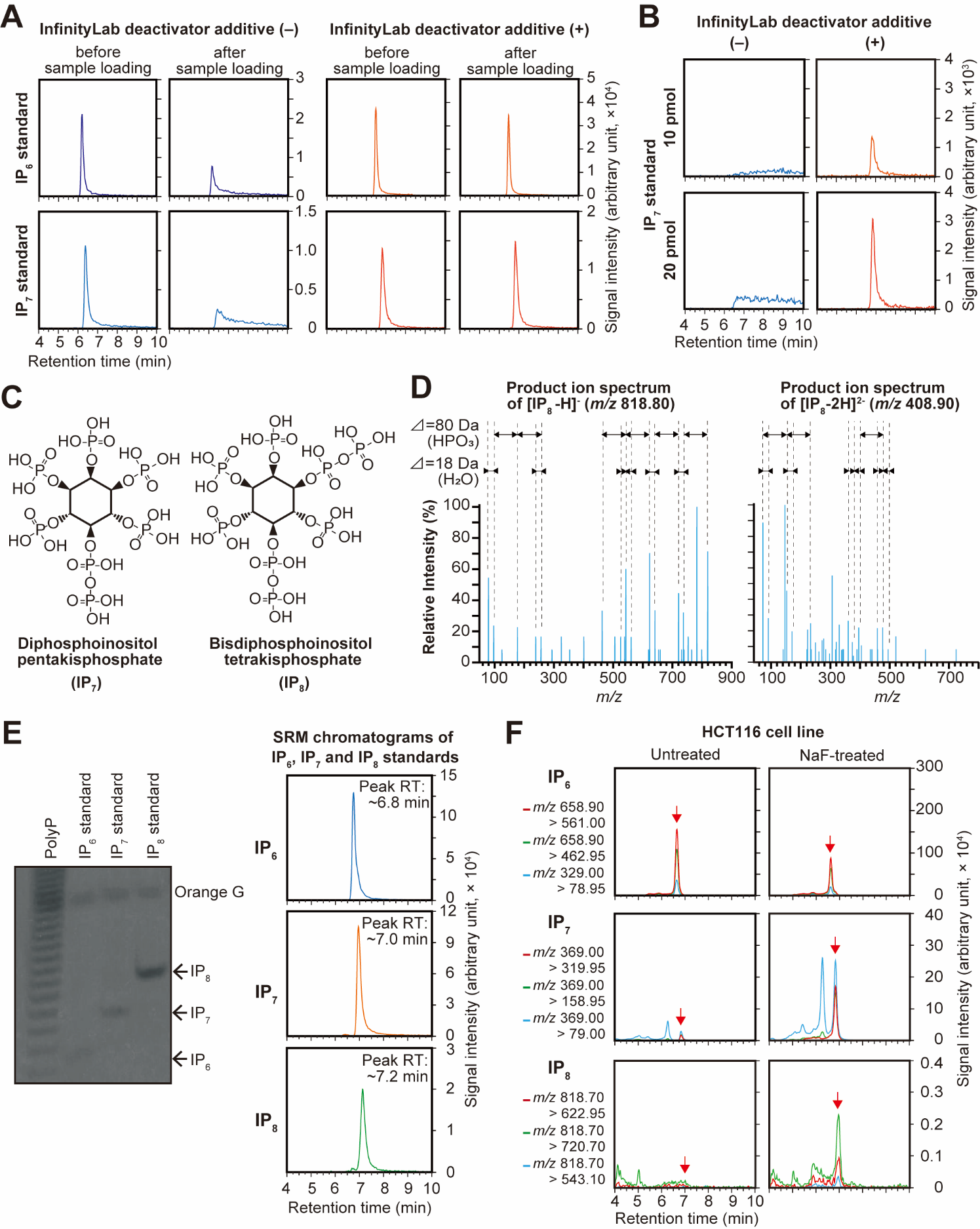
Supplementary Figure S1**

**Figure S1. Refined HILIC-MS/MS analysis for IP_6_ and PP-IPs. (A)** Effect of InfinityLab deactivator additive as a mobile phase modifier on SRM chromatograms of IP_6_ and IP_7_ before and after biological sample injection. 100 pmol of each synthetic analyte were injected. SRM chromatograms corresponding to IP_6_ (*m/z* 658.90 > 561.00) and IP_7_ (*m/z* 369.00 > 79.00) are depicted. **(B)** Effect of InfinityLab deactivator additive as a mobile phase modifier on the detection of low amounts of synthetic PP-IP. 10 and 20 pmol of IP_7_ standard were injected. SRM chromatograms corresponding to IP_7_ (*m/z* 369.00 > 79.00) are depicted. **(C)** Chemical structure of IP_7_ and IP_8_. **(D)** Product ion spectrum of IP_8_ (singly deprotonated precursor, left panel; doubly deprotonated precursor, right panel). Characteristic fragment ions generated by loss of water (H_2_O, 18 Da) and phosphoric acid (H_3_PO_4_, 80 Da) are also shown. **(E)** Gel electrophoretic results (left panel) and SRM chromatograms (right panel) of synthetic IP_6_, IP_7_, and IP_8_ standard. The PolyP ladder was used as an electrophoresis standard. 500 pmol of each standard were injected for LC-MS. SRM chromatograms corresponding to IP_6_ (*m/z* 658.90 > 561.00), IP_7_ (*m/z* 369.00 > 79.00), and IP_8_ (*m/z* 818.70 > 720.70) are depicted. **(F)** Representative SRM chromatograms of IP_6_, IP_7_, and IP_8_ in untreated (left panel) and NaF-treated (right panel) HCT116 cell samples. The three best transitions per molecule are shown for the peak identification of each compound. Arrows indicate the SRM peak of corresponding analytes. HILIC, hydrophilic interaction liquid chromatography; LC-MS, liquid chromatography-mass spectrometry; MS/MS, tandem mass spectrometry; NaF, sodium fluoride; PolyP, polyphosphate; PP-IP, inositol pyrophosphate; RT, retention time; SRM, selected reaction monitoring.

**Supplementary Figure S2**


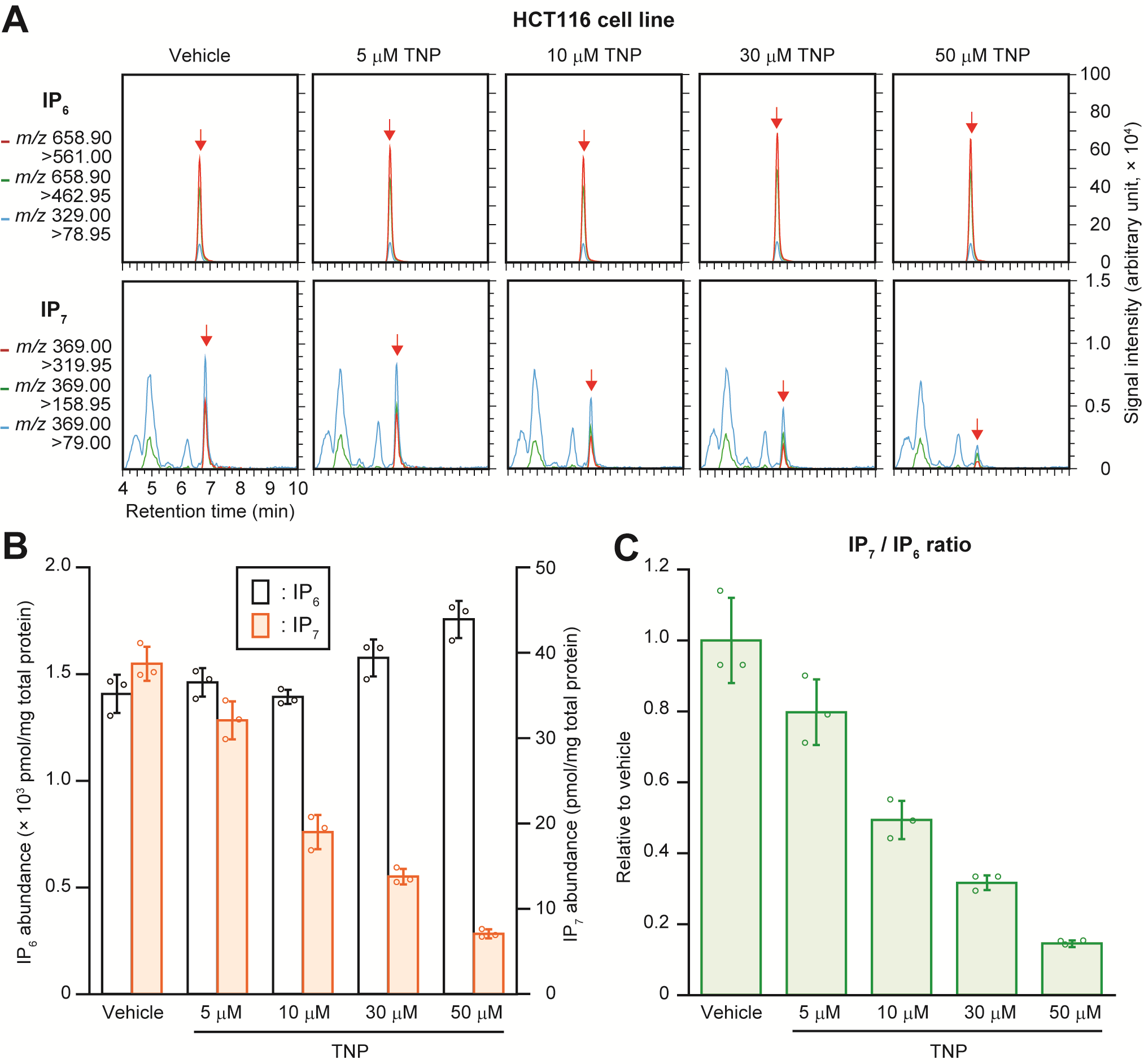


**Figure S2. HILIC-MS/MS protocol can detect dose-dependent reduction of IP_7_ production by IP6K inhibitor TNP in HCT116 cells.** **(A)** Representative SRM chromatograms of IP_6_ and IP_7_ in HCT116 cells treated for 1 h with different concentrations (0, 5, 10, 30, 50 μM) of the IP6K inhibitor TNP. Arrows indicate SRM peaks of corresponding analytes. **(B)** Concentrations of IP_6_ and IP_7_ in TNP-treated HCT116 cells. The values shown are expressed as pmol per mg of total protein (n = 3). **(C)** IP_7_/IP_6_ ratios in TNP-treated HCT116 cells. The values shown are expressed relative to those for vehicle-treated counterparts (n = 3). HILIC, hydrophilic interaction liquid chromatography; MS/MS, tandem mass spectrometry; SRM, selected reaction monitoring.

**Supplementary Figure S3**


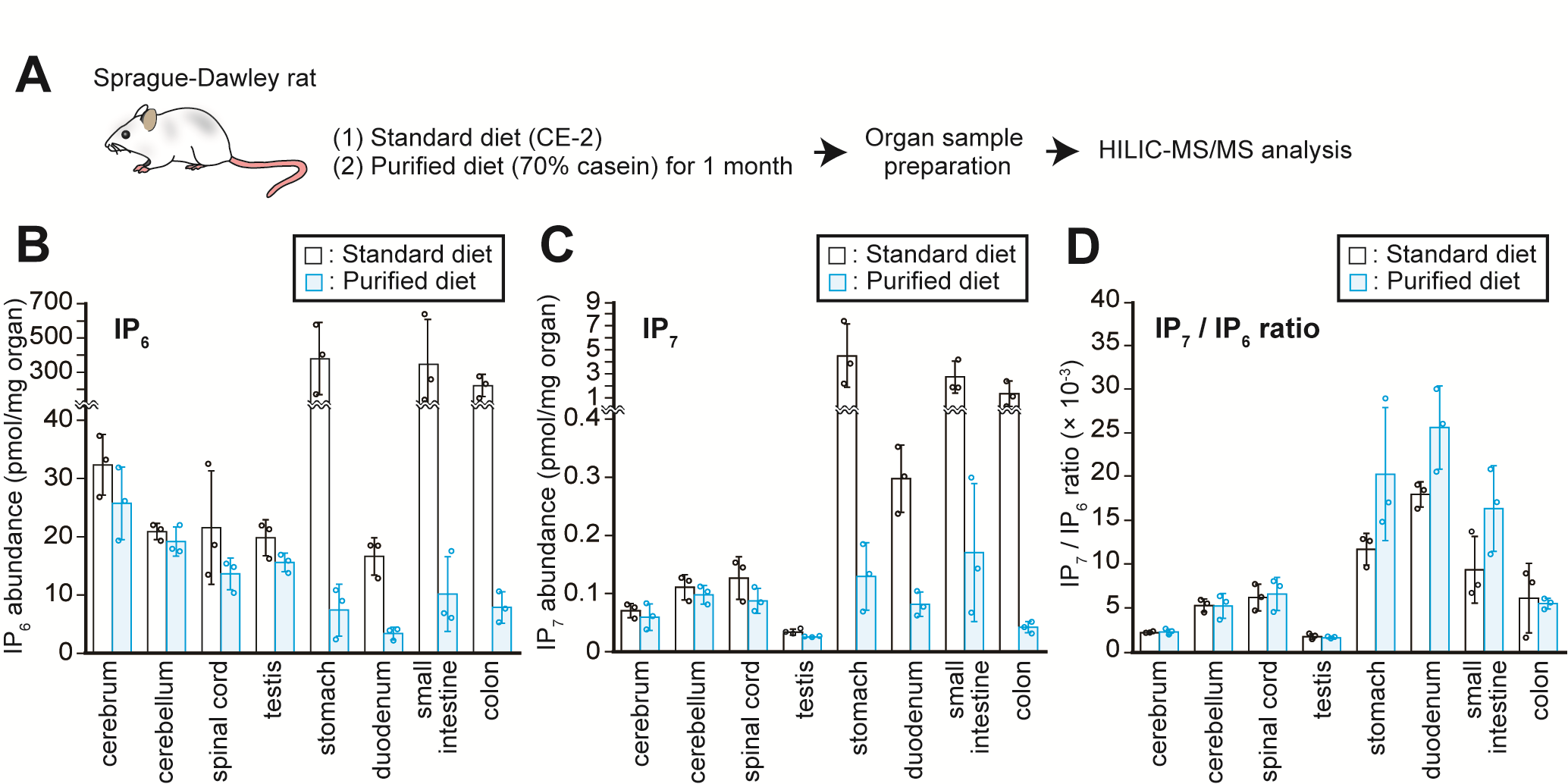


**Figure S3. IP_7_ metabolism is enhanced in the proximal GIT of Sprague-Dawley rats irrespective of dietary PP-IP supply. (A)** Graphical scheme of the experiment. Sprague–Dawley rats were fed a standard (n = 3) or purified diet (70% casein) for 1 month (n = 3). **(B-D)** Concentrations of IP_6_ (B), IP_7_ (C) and IP_7_/IP_6_ ratios (D) in the CNS, testes, and GIT of the male rats under the two different conditions. The values shown are expressed as pmol per mg of organ weight. CNS, central nervous system; GIT, gastrointestinal tract; PP-IP, inositol pyrophosphate.

**Supplementary Figure S4**


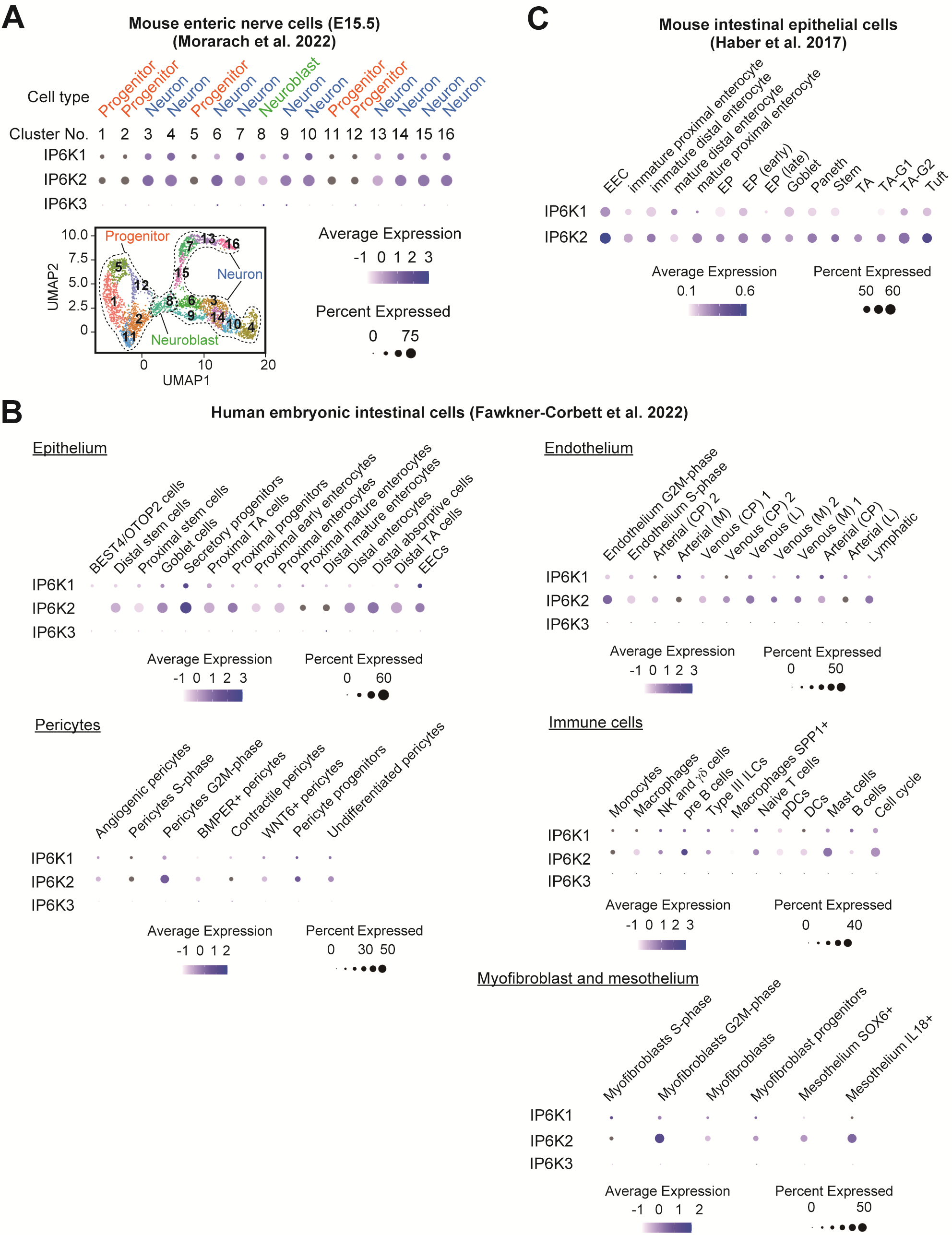


**Figure S4. IP6K-expressing enteric cell subsets obtained from public scRNA-seq datasets. (A)** UMAP-based unsupervised clustering of recently reported mouse embryonic (E15.5) ENS data (40). Assignment of cell identities was based on the expression of signature genes—Sox10 (Progenitor), Ascl1 (Neuroblast), Elavl4 (Enteric Neuron)—as described in the literature. Relative expression (log scale) of IP6K1 and IP6K2 among the ENS clusters. **(B)** Relative expression (log scale) of IP6K1 and IP6K2 in the subpopulation of human enteric cells excepting enteric neural cells, obtained by analysis of human prenatal intestinal scRNA-seq datasets (39). **(C)** Relative expression (log scale) of IP6K1 and IP6K2 in mouse intestinal epithelial cells, obtained by analysis of the corresponding scRNA-seq datasets (41). The size and color of the dots represent the percentage of cells that express IP6K1 and IP6K2 mRNAs and their average abundances within a cluster, respectively. CP, venous capillaries; DC, dendritic cell; E, embryonic day; EEC, enteroendocrine cell; ENS, enteric nervous system; EP, enterocyte progenitor; ILC, innate lymphoid cell; L, large sized; M, medium sized; NK, natural killer; scRNA-seq, single-cell RNA sequencing; TA, transit amplifying; UMAP, uniform manifold approximation and projection.

**Supplementary Figure S5**


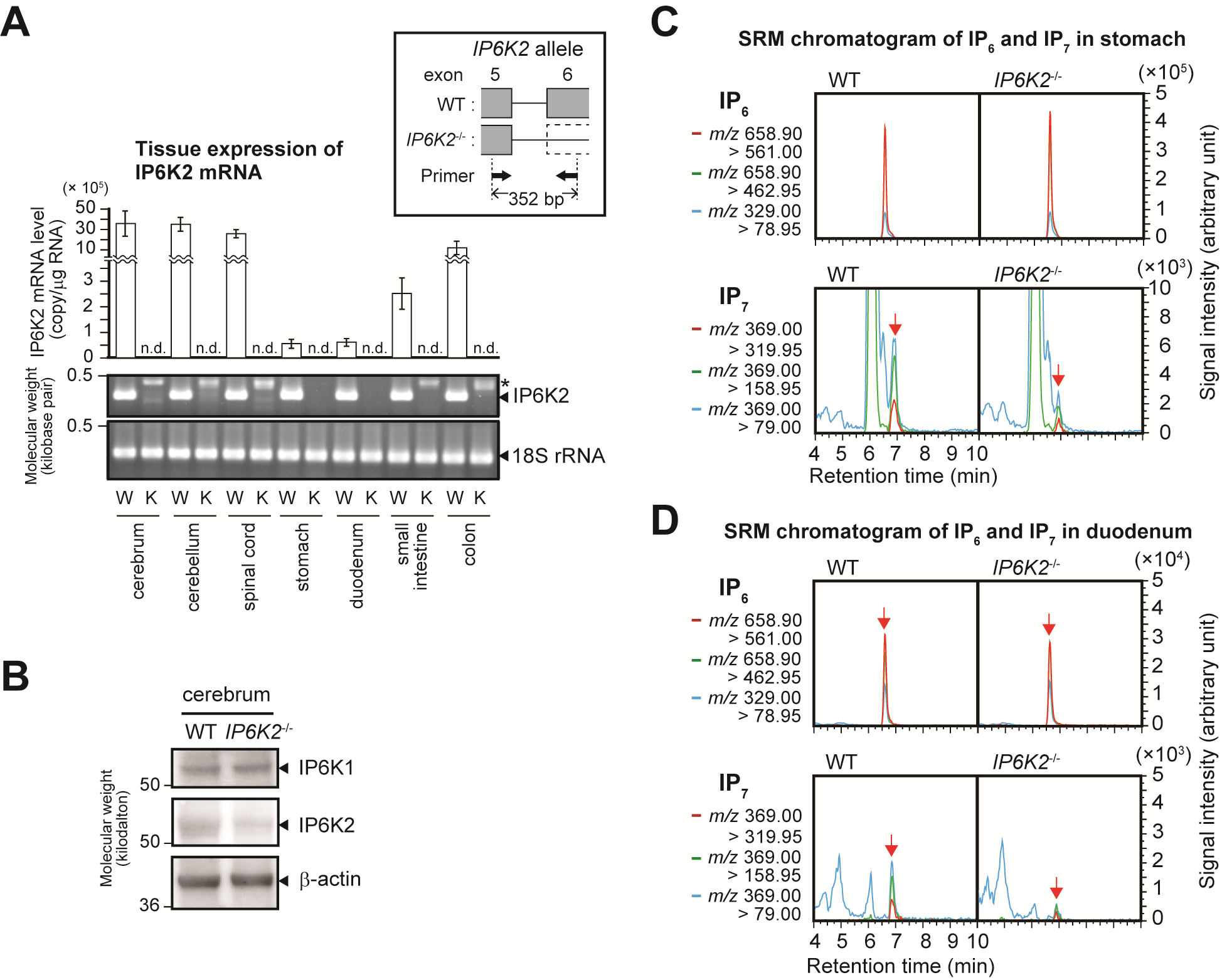


**Figure S5. HILIC-MS/MS analysis of IP_6_ and IP_7_ in the proximal GIT of IP6K2^-/-^ mice. (A)** IP6K2 mRNA levels in the CNS and GIT of IP6K2^-/-^ and WT mice. The values shown are normalized with 18S rRNA level and expressed as copies per μg RNA (n = 3). Electrophoretic gel images of qPCR products and PCR primer location in the IP6K2 genomic locus (upper right panel) was also depicted. W, WT; K, IP6K2^-/-^; n.d., not detected; *, non-specific band. **(B)** Representative Western blot image of IP6K1 and IP6K2 expression in the cerebrum of IP6K2^-/-^ and WT mice. β-actin was used as the internal control. **(C, D)** Representative SRM chromatograms of IP_6_ (upper panel) and IP_7_ (lower panel) in the stomach (C) and duodenum (D) of male IP6K2^-/-^ and WT mice. The three best transitions per molecule are shown for peak identification of each compound. Arrows indicate the SRM peak of each analyte. CNS, central nervous system; GIT, gastrointestinal tract; HILIC, hydrophilic interaction liquid chromatography; MS/MS, tandem mass spectrometry; SRM, selected reaction monitoring.

**Supplementary Figure S6**

**
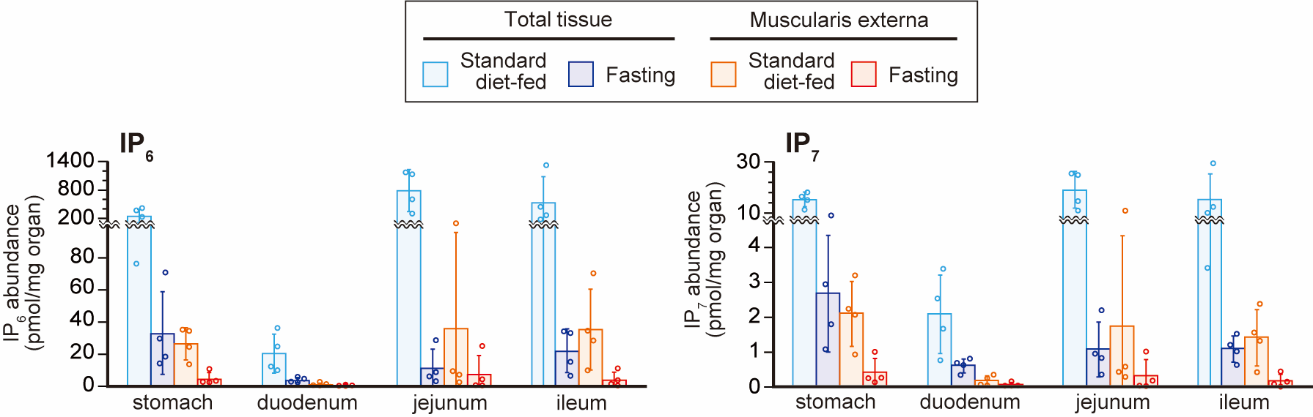
**

**Figure S6. The abundances of IP_6_ and IP_7_ in the muscularis externa and total tissue of four proximal GIT segments of C57BL/6J mice** **under the two different conditions.** The values shown represent the mean ± SD of four independent experiments and are expressed as pmol per mg of organ weight. GIT, gastrointestinal ract.

**Supplementary Figure S7**


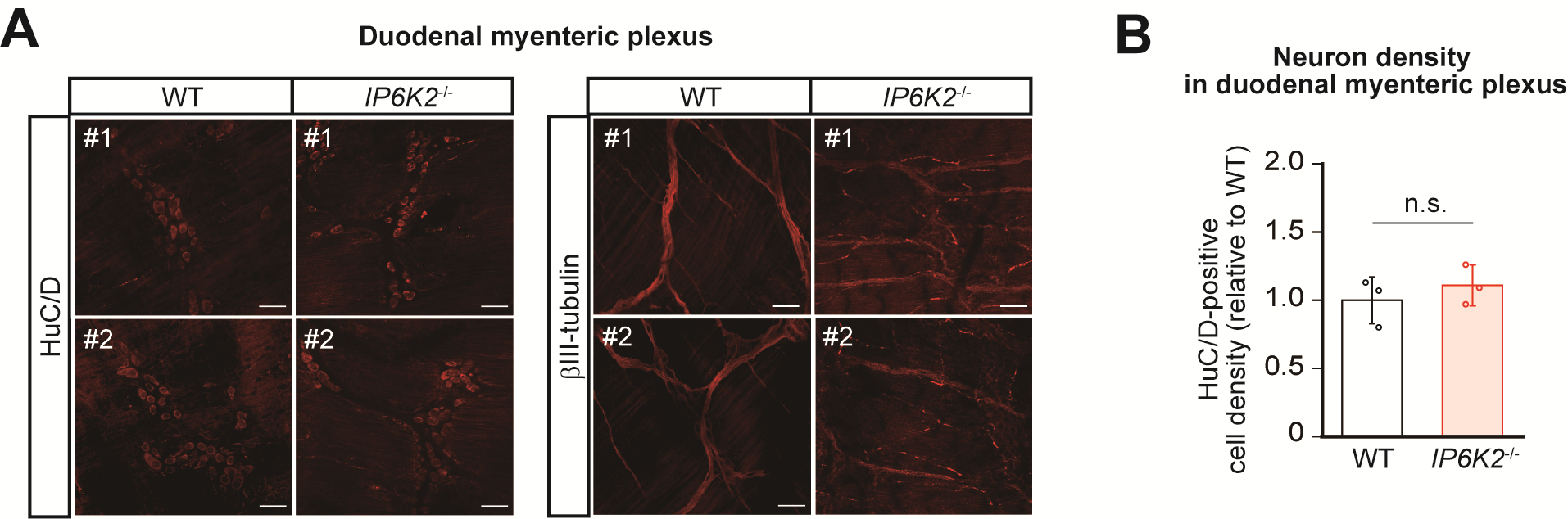


**Figure S7. IP6K2 inhibition does not affect morphological phenotype of the duodenal myenteric plexus. (A)** Whole mount immunostaining of WT and IP6K2^-/-^ duodenal muscularis externa using anti-neuronal markers antibodies. Two different areas of confocal microscopic images of each neuron marker are shown. The neuronal markers HuC/D and βIII-tubulin were detected to identify enteric neuronal somas and enteric nerve fibers in the myenteric plexuses, respectively. The scale bar represents 50 μm. **(B)** The concentration of enteric neurons in WT and IP6K2^-/-^ duodenal muscularis externa. The values shown represent the mean ± SD of three independent experiments and are expressed relative to those of WT mice. n.s., not significant (Student’s *t*-test).

**Supplementary Figure S8**


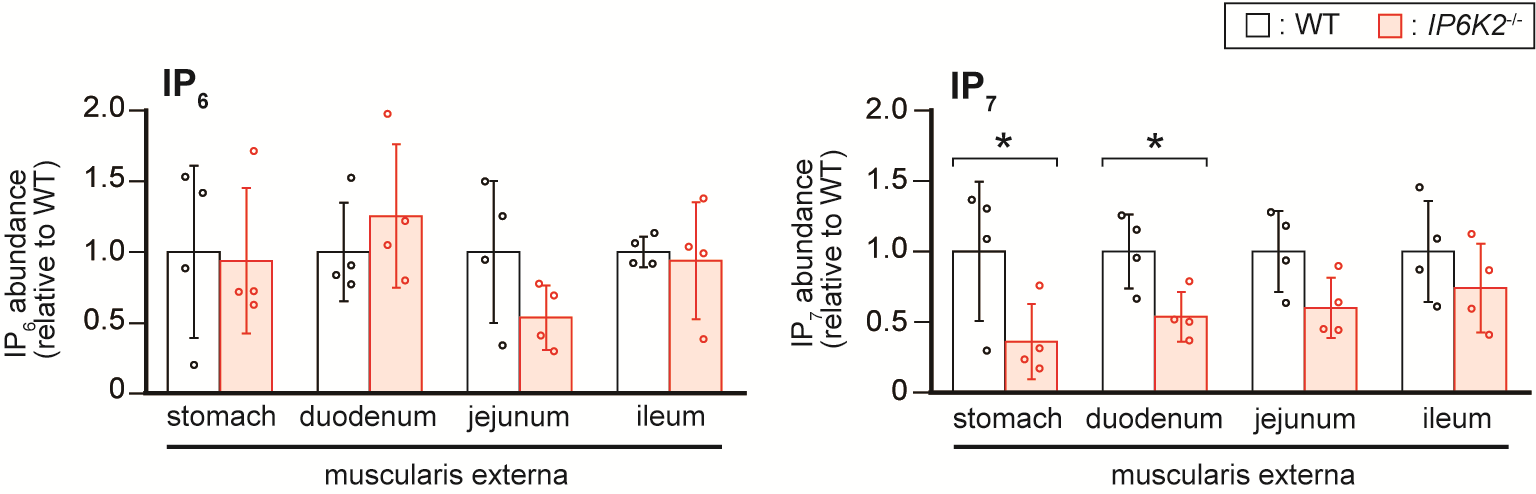


**Figure S8. The abundances of IP_6_ and IP_7_ in the muscularis externa of the four GIT segments of IP6K2^-/-^ and WT mice.** The values shown represent the mean ± SD of four independent experiments and are expressed relative to those for WT mice. Asterisks indicate statistical significance (*p* < 0.05, Student’s *t*-test) compared with WT mice. GIT, gastrointestinal tract.

**Supplementary Figure S9**


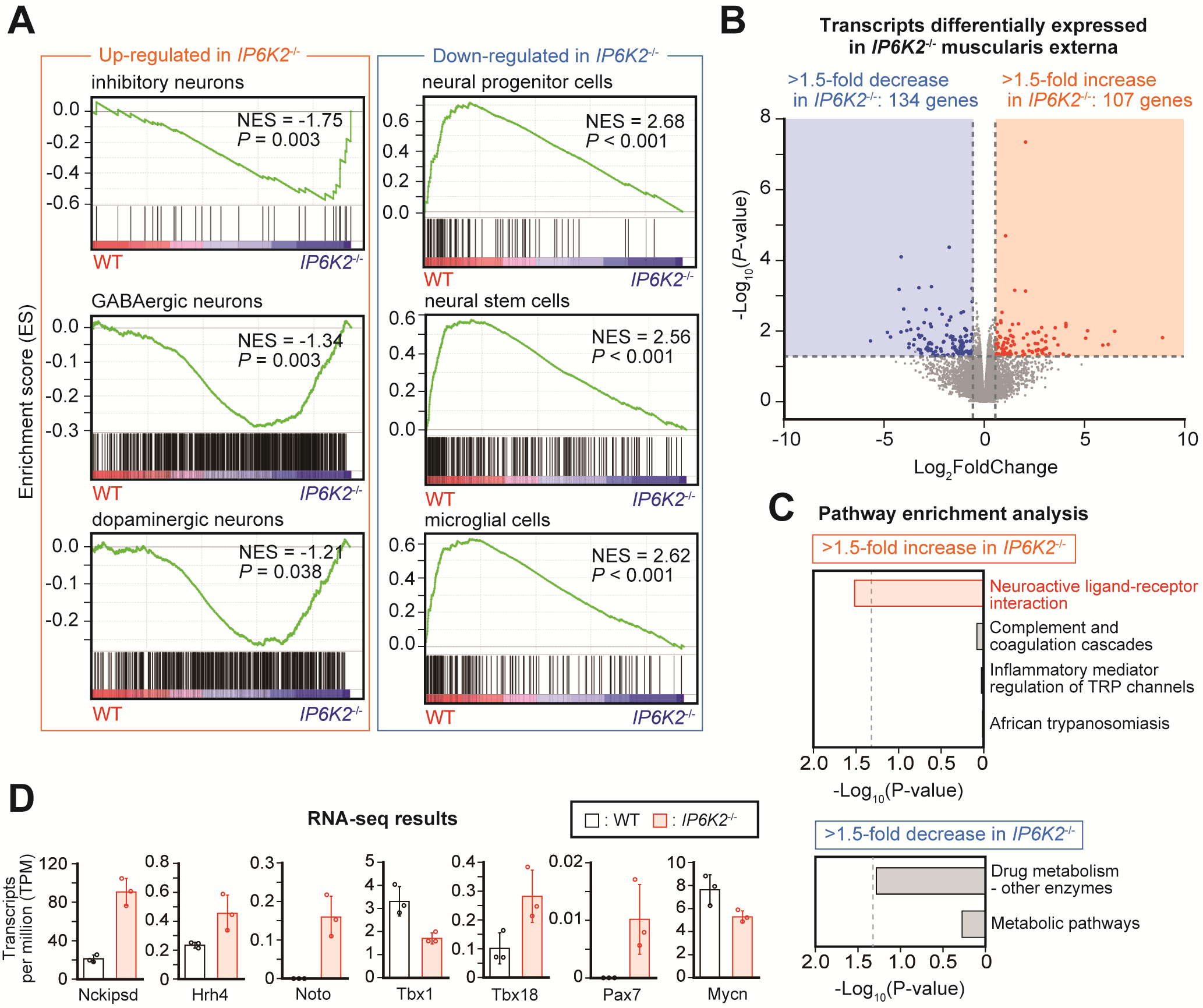


**Figure S9. RNA-seq analysis of transcripts differentially expressed in IP6K2^-/-^ duodenal muscularis externa. (A)** Representative GSEA plots of the gene sets enriched among up-regulated (inhibitory neurons, GABAergic neurons, dopaminergic neurons) or down-regulated (neural progenitor cells, neural stem cells, glial cells) genes by genetic ablation of IP6K2 in the duodenal muscularis externa. **(B)** Volcano plot showing transcripts differentially expressed in IP6K2^-/-^ duodenal muscularis externa compared with WT counterparts (n = 3). Horizontal dashed line indicates *p* value 0.05, and vertical lines indicate 1.5-fold cutoff. Transcripts enriched or depleted more than 1.5-fold with *p* value < 0.05 by IP6K2 deletion were labeled in red or blue dots, respectively. **(C)** Pathway enrichment analysis for transcripts with more than 1.5-fold enrichment or depletion with *p* value < 0.05 in the duodenum muscularis externa of IP6K2^-/-^ mice compared with WT counterparts. KEGG pathways influenced by IP6K2 inhibition are shown. Pathways associated with neuronal regulations are highlighted in red. Vertical dashed lines indicate *p* value 0.05. **(D)** Normalized expression levels (transcripts per million, TPM) from RNA-seq data for 7 neuronal genes prominently and significantly (*p* < 0.05, Student’s *t*-test) accumulated or depleted in IP6K2^-/-^ duodenal muscularis externa compared with WT counterparts (n = 3). GSEA, gene set enrichment analysis; KEGG, Kyoto encyclopedia of genes and genomes; RNA-seq, RNA sequencing.

**Supplementary Figure S10**


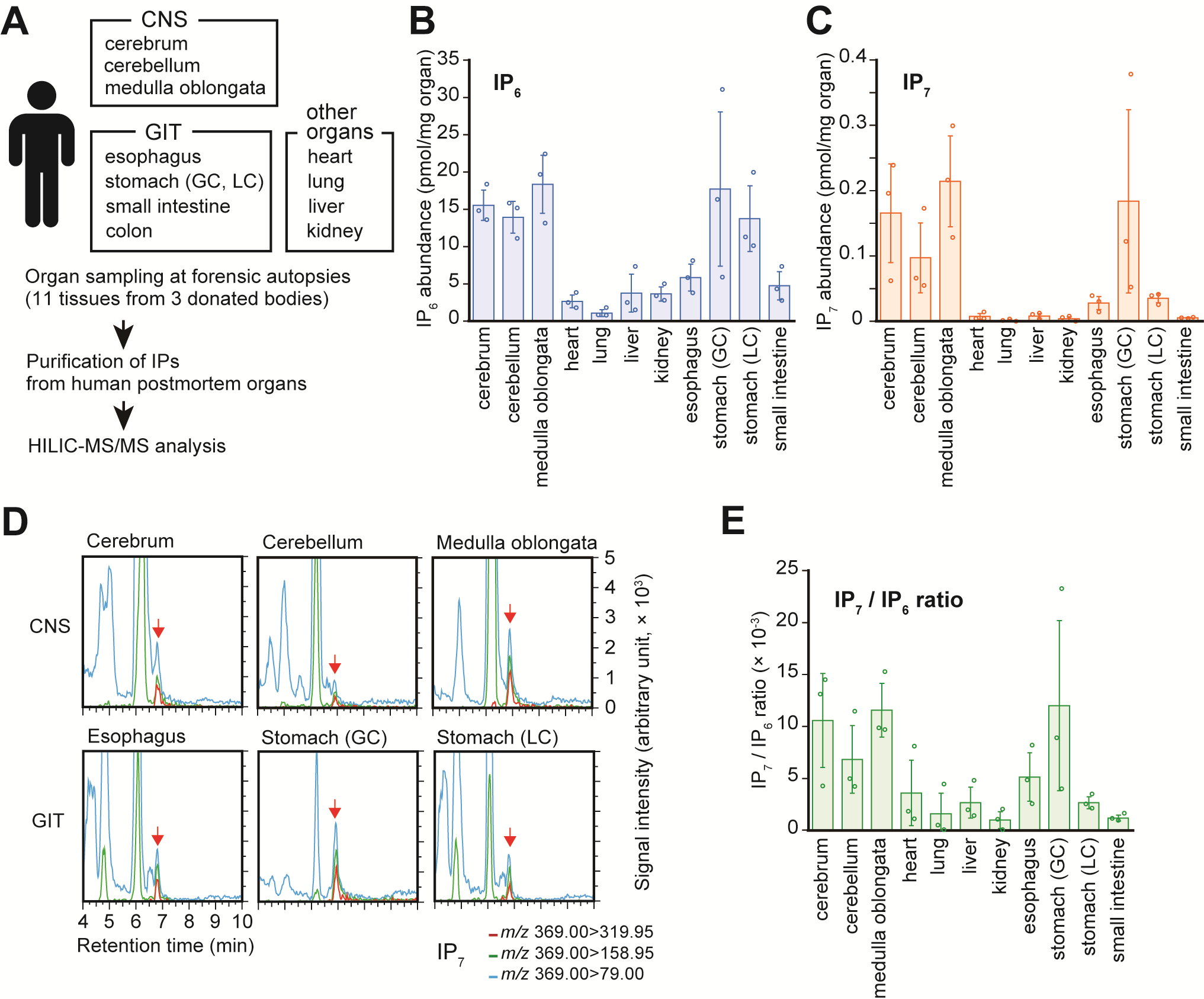


**Figure S10. HILIC-MS/MS protocol can detect IP_7_ in human postmortem organs. (A)** Schematic workflow of human organ analysis. Human postmortem organs were obtained after autopsies in three forensic cases. **(B, C)** The concentration of IP_6_ (B) and IP_7_ (C) in human postmortem organs. The values shown are expressed as pmol per mg of organ weight (n = 3). **(D)** Representative SRM chromatograms of IP_7_ in human postmortem CNS (cerebrum, cerebellum, and medulla oblongata) and proximal GIT (esophagus, greater curvature and lesser curvature of stomach) samples. The three best transitions are shown for IP_7_ peak identification. Arrows indicate SRM peak of IP_7_. **(E)** IP_7_/IP_6_ ratios in human postmortem organs (n = 3). CNS, central nervous system; GC, greater curvature; GIT, gastrointestinal tract; HILIC, hydrophilic interaction liquid chromatography; LC, lesser curvature; MS/MS, tandem mass spectrometry; SRM, selected reaction monitoring.

**References**

39. D.​ ​​Fawkner-Corbett​, ​​​A.​ ​​Antanaviciute​, ​​​K.​ ​​Parikh​, ​​​M.​ ​​Jagielowicz​, ​​​A.S.​ ​​Gerós​, ​​​T.​ ​​Gupta​, ​​et al.​, ​​Spatiotemporal analysis of human intestinal development at single-cell resolution​, *​​Cell*​, ​​**184**​, ​​2021​, ​​810​–​​826.e23​.

40. ​​​K.​ ​​Morarach​, ​​​A.​ ​​Mikhailova​, ​​​V.​ ​​Knoflach​, ​​​F.​ ​​Memic​, ​​​R.​ ​​Kumar​, ​​​W.​ ​​Li​, ​​et al.​, ​​Diversification of molecularly defined myenteric neuron classes revealed by single-cell RNA sequencing​, ​​*Nat. Neurosci*.​, ​​**24**​, ​​2021​, ​​34​–​​46​.

41. A.L.​ ​​Haber​, ​​​M.​ ​​Biton​, ​​​N.​ ​​Rogel​, ​​​R.H.​ ​​Herbst​, ​​​K.​ ​​Shekhar​, ​​​C.​ ​​Smillie​, ​​et al.​, ​​A single-cell survey of the small intestinal epithelium​, ​​*Nature*​, ​​**551**​, ​​2017​, ​​333​–​​339​.
