# Supplementary material for: Inositol pyrophosphate profiling reveals regulatory roles of IP6K2-dependent enhanced IP7 metabolism in the enteric nervous system
Source: J Biol Chem. 2023 Jan 19;299(3):102928. doi: 10.1016/j.jbc.2023.102928 (PMC9957762; doi:10.1016/j.jbc.2023.102928)
Supplement: Supporting information tables [file mmc2.docx]

Supplementary Tables for:

**Inositol pyrophosphate profiling reveals regulatory roles of IP6K2-dependent enhanced IP_7_ metabolism**

**in the enteric nervous system**

Running title: IP6K2-IP_7_ axis regulates the enteric nervous system

Masatoshi Ito^1,*^, Natsuko Fujii^2^, Saori Kohara^2^, Shuho Hori^1^, Masayuki Tanaka^1^, Christopher Wittwer^3^, Kenta Kikuchi^4^, Takatoshi Iijima^5^, Yu Kakimoto^6^, Kenichi Hirabayashi^7^, Daisuke Kurotaki^4^, Henning J. Jessen^3^, Adolfo Saiardi^8^, Eiichiro Nagata^2,*^

^1^Support Center for Medical Research and Education, Tokai University, Isehara, Japan; ^2^Department of Neurology, Tokai University School of Medicine, Isehara, Japan; ^3^Institute of Organic Chemistry, University of Freiburg, Freiburg, Germany; ^4^Laboratory of Chromatin Organization in Immune Cell Development, International Research Center for Medical Sciences, Kumamoto University, Kumamoto, Japan; ^5^Department of Molecular Life Science, ^6^Department of Forensic Medicine, and ^7^Department of Pathology, Tokai University School of Medicine, Isehara, Japan; ^8^Medical Research Council Laboratory for Molecular Cell Biology, University College London, London, United Kingdom

* For correspondence:

Masatoshi Ito, [masa104-ito@tokai-u.jp](mailto:masa104-ito@tokai-u.jp); Eiichiro Nagata, [enagata@is.icc.u-tokai.ac.jp](mailto:enagata@is.icc.u-tokai.ac.jp)

Present address for Masatoshi Ito: Department of Legal Medicine at St. Marianna University School of Medicine, Kawasaki, Kanagawa 216-8511, Japan; e-mail: [masatoshi.ito@marianna-u.ac.jp](mailto:masatoshi.ito@marianna-u.ac.jp)

**Supplementary Table S1. Optimal SRM conditions for IP_8_ detection using HILIC-MS/MS.**

| **Transition** | **Q1prebias (V)** | **Collision energy (V)** | **Q3prebias (V)** |
| --- | --- | --- | --- |
| 818.70 > 622.95 | 22 | 33 | 20 |
| 818.70 > 720.70 | 30 | 27 | 24 |
| 818.70 > 543.10 | 40 | 45 | 40 |

**Supplementary Table S2. The concentrations of IP_6_, IP_7_ and IP_7_/IP_6_ ratios in the 15 organs of standard diet-fed C57BL/6J mice.**

|  |  | **cerebrum** | **cerebellum** | **spinal cord** | **heart** | **lung** | **liver** | **kidney** | **pancreas** | **spleen** | **gastro**  **cnemius** | **testis** | **stomach** | **duodenum** | **small intestine** | **colon** |
| --- | --- | --- | --- | --- | --- | --- | --- | --- | --- | --- | --- | --- | --- | --- | --- | --- |
| **IP_6_ (pmol/mg organ)** | **#1** | 25.62 | 18.56 | 15.49 | 6.06 | 21.14 | 76.27 | 36.68 | 23.81 | 18.10 | 0.83 | 11.65 | 75.16 | 7.83 | 562.64 | 270.54 |
|  | **#2** | 28.87 | 16.10 | 14.37 | 5.39 | 17.21 | 45.60 | 25.19 | 26.11 | 22.36 | 1.37 | 9.69 | 132.20 | 9.26 | 148.47 | 122.52 |
|  | **#3** | 32.24 | 14.19 | 15.98 | 5.17 | 21.04 | 46.74 | 31.95 | 56.19 | 18.46 | 1.04 | 9.57 | 113.44 | 44.50 | 100.46 | 345.70 |
|  | **#4** | 17.49 | 12.48 | 14.46 | 3.23 | 10.62 | 48.83 | 29.35 | 34.01 | 16.46 | 2.44 | 9.71 | 97.19 | 8.83 | 233.85 | 34.52 |
|  | **average** | 26.06 | 15.33 | 15.08 | 4.96 | 17.50 | 54.36 | 30.79 | 35.03 | 18.85 | 1.42 | 10.15 | 104.50 | 17.60 | 261.35 | 193.32 |
|  | **s.d.** | 6.32 | 2.61 | 0.79 | 1.22 | 4.94 | 14.67 | 4.81 | 14.77 | 2.50 | 0.72 | 1.00 | 24.23 | 17.94 | 208.29 | 140.73 |
| **IP_7_ (pmol/mg organ)** | **#1** | 0.214 | 0.187 | 0.300 | 0.024 | 0.120 | 0.465 | 0.089 | 0.281 | 0.066 | 0.001 | 0.050 | 9.145 | 1.257 | 13.755 | 7.606 |
|  | **#2** | 0.395 | 0.288 | 0.209 | 0.035 | 0.310 | 0.206 | 0.164 | 0.560 | 0.413 | 0.000 | 0.028 | 14.751 | 1.735 | 6.724 | 2.786 |
|  | **#3** | 0.349 | 0.293 | 0.521 | 0.012 | 0.239 | 0.232 | 0.229 | 0.794 | 0.117 | 0.000 | 0.053 | 12.187 | 4.231 | 8.464 | 9.284 |
|  | **#4** | 0.267 | 0.229 | 0.350 | 0.000 | 0.085 | 0.297 | 0.061 | 0.188 | 0.071 | 0.020 | 0.047 | 8.620 | 1.480 | 12.641 | 1.797 |
|  | **average** | 0.306 | 0.249 | 0.345 | 0.018 | 0.188 | 0.300 | 0.136 | 0.455 | 0.167 | 0.006 | 0.045 | 11.176 | 2.176 | 10.396 | 5.368 |
|  | **s.d.** | 0.081 | 0.051 | 0.131 | 0.015 | 0.104 | 0.116 | 0.076 | 0.275 | 0.166 | 0.010 | 0.011 | 2.856 | 1.384 | 3.343 | 3.640 |
| **IP_7_/IP_6_ ratio (× 10^-3^)** | **#1** | 8.34 | 10.06 | 19.34 | 3.90 | 5.67 | 6.09 | 2.43 | 11.80 | 3.65 | 1.77 | 4.31 | 121.67 | 160.56 | 24.45 | 28.11 |
|  | **#2** | 13.68 | 17.90 | 14.51 | 6.53 | 17.99 | 4.51 | 6.50 | 21.44 | 18.45 | 0.00 | 2.87 | 111.59 | 187.29 | 45.29 | 22.74 |
|  | **#3** | 10.83 | 20.63 | 32.61 | 2.30 | 11.37 | 4.96 | 7.15 | 14.12 | 6.34 | 0.44 | 5.57 | 107.43 | 95.08 | 84.25 | 26.86 |
|  | **#4** | 15.28 | 18.37 | 24.22 | 0.00 | 8.01 | 6.08 | 2.08 | 5.52 | 4.30 | 8.28 | 4.84 | 88.69 | 167.58 | 54.06 | 52.07 |
|  | **average** | 12.03 | 16.74 | 22.67 | 3.18 | 10.76 | 5.41 | 4.54 | 13.22 | 8.19 | 2.63 | 4.40 | 107.34 | 152.63 | 52.01 | 32.45 |
|  | **s.d.** | 3.07 | 4.61 | 7.72 | 2.75 | 5.36 | 0.80 | 2.66 | 6.57 | 6.94 | 3.85 | 1.14 | 13.80 | 40.00 | 24.82 | 13.28 |

**Supplementary Table S3. The concentrations of IP_6_, IP_7_ and relative amount of IP_8_ in experimental rodent diets.**

|  | **IP_6_ (nmol/mg)** | **IP_7_ (nmol/mg)** | **IP_8_ (relative to Standard diet)** |
| --- | --- | --- | --- |
| **Standard diet**  **(CE-2)** | **3.96 ± 0.82** | **0.17 ± 0.03** | **1.00 ± 0.30** |
| **Purified diet**  **(iVid-neo)** | **0.27 ± 0.04** | **< 0.01** | **0.01 ± 0.01** |
| **Purified diet**  **(70% casein)** | **< 0.01** | **< 0.01** | **< 0.01** |

**Supplementary Table S4. DNA primers used for qPCR analysis in this study.**

| **Gene symbol** | **Accession** | **Official name** | **Direction** | **Sequence (5’ - 3’)** |
| --- | --- | --- | --- | --- |
| Ip6k2 | NM_029634.2 | inositol hexakisphosphate kinase 2 | Forward | GAACCTGACTTCCCGCTATG |
|  |  |  | Reverse | GTAGGATTCCTGTCGCTCCA |
| Drd5 | NM_013503.3 | dopamine receptor D5 | Forward | ACCAAGACACGGTCTTCCAC |
|  |  |  | Reverse | CCTCCTCCTCACAGTCAAGC |
| Cckbr | NM_007627.5 | cholecystokinin B receptor | Forward | TCTCCCGCGAACTCTACCTA |
|  |  |  | Reverse | CAGCGTTGTCATCTCCAGTC |
| Npy4r | NM_008919.4 | neuropeptide Y receptor Y4 | Forward | CCATGGCAACCTCATCTTCT |
|  |  |  | Reverse | TCATCGATCCCTTGGATAGG |
| Nckipsd | NM_030729.4 | NCK interacting protein with SH3 domain | Forward | CCGCTGCTATCTGGAAGAAC |
|  |  |  | Reverse | AGCACGGAAGACACCAGAGT |
| Noto | NM_001007472.2 | notochord homeobox | Forward | AATGTCACTCACCACCAGCA |
|  |  |  | Reverse | CAGCTGGGCTCTCTCCTTC |
| Tbx1 | NM_011532.2 | T-box 1 | Forward | TGAGGAGACACGCTTCACTG |
|  |  |  | Reverse | CTGCAGCGTCTTTGTCTGAG |
| Hrh4 | NM_153087.2 | histamine receptor H4 | Forward | AGCCTTTGTGGTGGACAGAA |
|  |  |  | Reverse | TCGATCGTAGCTAATGAGGACA |
| Tbx18 | NM_023814.4 | T-box18 | Forward | GGATTAGACCCTCACCAGCA |
|  |  |  | Reverse | CCTTGGTCATCCAGCTCATT |
| Pax7 | NM_011039.2 | paired box 7 | Forward | TACCAGCTGCCGGACTCTAC |
|  |  |  | Reverse | TGACAGGGTTCATGTGGTTG |
| Mycn | NM_008709.3 | v-myc avian myelocytomatosis viral related oncogene, neuroblastoma derived | Forward | GCTGCGGTCACTAGTGTGTC |
|  |  |  | Reverse | AAGTGGTTACCGCCTTGTTG |
| Actb | NM_007393.5 | actin, beta | Forward | CATGAAGTGTGACGTTGACATC |
|  |  |  | Reverse | ATGATCTTGATCTTCATGGTGC |
| Rn18S | NR_003278.3 | 18S ribosomal RNA | Forward | GTAACCCGTTGAACCCCATT |
|  |  |  | Reverse | AGTTCGACCGTCTTCTCAGC |
